# The mitochondrial ubiquitin ligase MARCH5 resolves MAVS aggregates during antiviral signalling

**DOI:** 10.1038/ncomms8910

**Published:** 2015-08-06

**Authors:** Young-Suk Yoo, Yong-Yea Park, Jae-Hoon Kim, Hyeseon Cho, Song-Hee Kim, Ho-Soo Lee, Tae-Hwan Kim, You Sun Kim, Youngsoo Lee, Chul-Joong Kim, Jae U Jung, Jong-Soo Lee, Hyeseong Cho

**Affiliations:** 1 Department of Biochemistry, Ajou University School of Medicine, Graduate School of Ajou University, Suwon 443-380, Korea; 2 Department of Biological Sciences, Graduate School of Ajou University, Suwon 443-380, Korea; 3 College of Veterinary Medicine (BK21 Plus Program), Chungnam National University, Daejeon 305-764, Korea; 4 Mucosal Immunobiology Section, Laboratory of Molecular Immunology, NIAID, NIH, Bethesda, Maryland 20852, USA; 5 Genomic Instability Research Center, Ajou University School of Medicine, Suwon 443-380, Korea; 6 Department of Molecular Microbiology and Immunology, Keck School of Medicine, University of Southern California, Los Angeles, California 90089, USA

## Abstract

Mitochondria serve as platforms for innate immunity. The mitochondrial antiviral signalling (MAVS) protein forms aggregates that elicit robust type-I interferon induction on viral infection, but persistent MAVS signalling leads to host immunopathology; it remains unknown how these signalling aggregates are resolved. Here we identify the mitochondria-resident E3 ligase, MARCH5, as a negative regulator of MAVS aggregates. *March5*^+/−^ mice and MARCH5-deficient immune cells exhibit low viral replication and elevated type-I interferon responses to RNA viruses. MARCH5 binds MAVS only during viral stimulation when MAVS forms aggregates, and these interactions require the RING domain of MARCH5 and the CARD domain of MAVS. MARCH5, but not its RING mutant (MARCH5^H43W^), reduces the level of MAVS aggregates. MARCH5 transfers ubiquitin to Lys7 and Lys500 of MAVS and promotes its proteasome-mediated degradation. Our results indicate that MARCH5 modulates MAVS-mediated antiviral signalling, preventing excessive immune reactions.

## Abstract (web summary)

RNA viral infections trigger an immune response mediated by the formation of aggregates of the MAVS protein. Here the authors show that the mitochondrial protein MARCH5 modulates this response by transferring ubiquitin to MAVS aggregates, thus promoting their proteasomal degradation.

## Graphical abstract

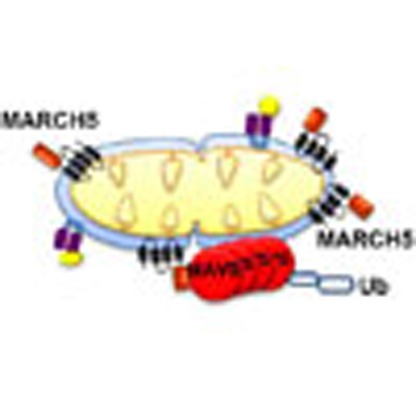

MARCH5 belongs to the MARCH family of membrane-bound E3 ubiquitin ligases, which are localized in the plasma membrane and in membranes of intracellular organelles such as endosomes, the endoplasmic reticulum and mitochondria^1^,^2^. MARCH family members are mammalian homologues of viral E3 ligases, which are modulators of immune recognition; in particular, they regulate the expression of cell-surface molecules such as major histocompatibility complex proteins and B7-2, a co-stimulatory molecule important for antigen presentation^2^,^3^,^4^,^5^,^6^,^7^. To date, however, the specific immune modulatory functions of the MARCH family in organelles remain unknown. MARCH5 (also known as MITOL or RNF153) is the only member of the MARCH family that resides on the outer mitochondrial membrane^8^. MARCH5 plays an important role in mitochondrial homeostasis, regulating mitochondrial dynamics via ubiquitination of the mitochondria fission and fusion proteins, Drp1 and mitofusins^9^,^10^,^11^,^12^, and disruption of the MARCH5-mediated mitochondria dynamics leads to cellular senescence or promotes cell death^11^,^12^. In addition, MARCH5 is also involved in protein quality control: it specifically recognizes and binds to mutated superoxide dismutase 1 and expanded polyglutamine aggregates that accumulated in mitochondria. MARCH5 targets them for degradation via the ubiquitin–proteasome pathway^13^,^14^,^15^,^16^. Although the underlying mechanisms by which MARCH5 specifically recognizes denatured or aggregated proteins remain unknown, its characteristics suggest that it is an important quality-control factor that makes a critical contribution to mitochondrial homeostasis.

The mitochondrial antiviral signalling (MAVS) protein plays an essential role in the innate immune response to RNA viruses. RIG-I-like receptors (RLRs), RIG-I, MDA5 and LGP2 are cytoplasmic RNA sensors that detect RNA derived from RNA viruses^17^,^18^,^19^,^20^. Although these sensors exhibit different ligand specificities for viral RNA, they all share MAVS (also known as VISA, Cardif or IPS-I) as a common adaptor protein that links RLRs to their downstream signalling molecules. MAVS forms well-ordered prion-like aggregates^21^,^22^,^23^; these aggregates are potent activators of NF-κB, IRF3 and IRF7 cascades, which result in production of type-I interferon (IFNI) and proinflammatory cytokines^24^,^25^,^26^. Although antiviral signalling generated from the RLRs is necessary to limit the spread of viral infection, attenuation of antiviral signalling is also essential for prevention of excessive production of IFNI and proinflammatory cytokines, which could cause deleterious effects on the host^27^,^28^,^29^,^30^,^31^,^32^,^33^. Consequently, host cells have developed several strategies that halt MAVS signalling, and a number of MAVS regulators in the cytoplasm have been identified. UBXN1-mediated interference with intracellular MAVS oligomerization^32^ and miniMAVS, a truncated variant of MAVS generated by alternative translation initiation, attenuate MAVS-mediated immune responses^34^. PCBP2 (ref. 28) and Ndfip1 (ref. 31) facilitate the degradation of MAVS by recruiting the cytosolic E3 ligases AIP4 and Smurf1, respectively. PSMA7 also induces MAVS degradation, although the molecular mechanism remains to be determined^29^. Importantly, MAVS is strategically localized to mitochondria, which serve as critical platforms for MAVS activation and propagation. To date, no other MAVS regulators have been proposed to target MAVS aggregates at mitochondria.

Here we demonstrate that under viral stimulation, mitochondria-resident MARCH5 targets MAVS aggregates and functions as an E3 ubiquitin ligase, promoting K48-linked ubiquitination-mediated degradation of MAVS. This MARCH5–MAVS interaction and degradation of MAVS aggregates at mitochondrial membranes contribute to the cellular homeostasis by preventing host immunopathology.

## Results

### *March5* ^+/−^ mice exhibited reduced morbidity

**Figure 1:**
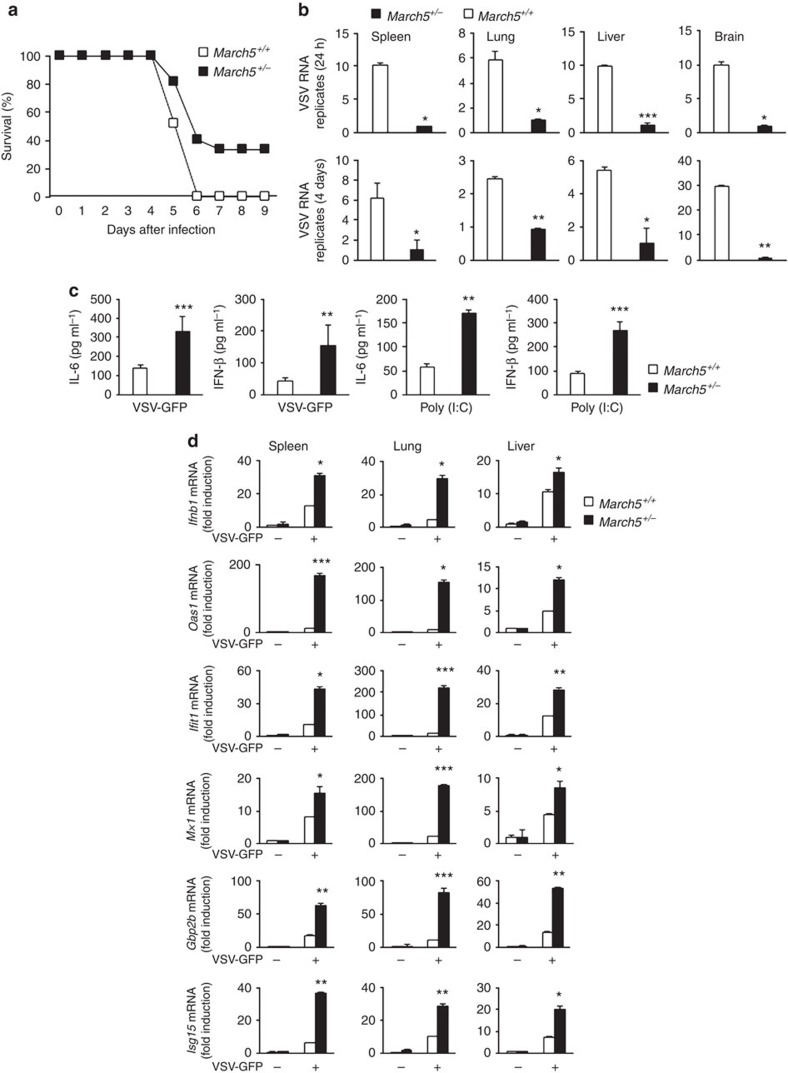
*March5*^+/−^ mice exhibited low viral replication and reduced morbidity in response to RNA viral infection. (**a**) Survival rate (%) of mice. *March5*^+/+^ and *March5*^+/−^ mice (6–7 weeks of age) were infected with VSV (2 × 10^8^ p.f.u. per mouse) via tail vein injection. Mortality was monitored for 9 days (*n*=15 mice per group). (**b**) VSV replication analysis using qPCR in *March5*^+/+^ and *March5*^+/−^ mouse tissues. *March5*^*+/+*^ and *March5*^*+/−*^ mice were infected with VSV; 4 days later, tissues from the spleen, lung, liver and brain were collected and analysed for VSV RNA replicates using qPCR. Error bars, mean±s.e.m. (*n*=9 mice per group). (**c**) Bioassay of IFN-β or IL-6 production in serum of *March5*^*+/+*^ and *March5*^*+/−*^ mice 12 h after infection with VSV (left, 2 × 10^8^ p.f.u. per mouse) or 200 μg of poly(I:C) injection (right) via the tail vein. Error bars, mean±s.e.m. (*n*=9 mice per group). (**d**) Quantitative PCR analysis of *Ifnb1*, *Oas1*, *Ifit1*, *Mx1*, *Gbp2b* and *Isg15* mRNA expression in the spleen, lung and liver of *March5*^*+/+*^ and *March5*^*+/−*^ mice 4 days after VSV infection. Graphs represent fold-induction of indicated genes relative to mRNA levels in *March5*^+/+^. Error bars, mean±s.e.m. (*n*=3 mice per group). *P<0.05, **P<0.01, ***P<0.001 versus *March5*^+/+^ by Student's *t*-test. All data are representative of at least three independent experiments.

Mitochondria serve as platforms for innate immune responses. Preliminary data obtained by streptavidin-affinity purification of MARCH5 suggested that MAVS is a candidate MARCH5-interacting protein. MAVS is a key molecule in the innate immune response against RNA viruses; hence, we investigated whether MARCH5 exerted any immune modulatory function in innate immunity. Because homozygous deletion of *March5* is embryonically lethal, we generated heterozygous *March5*^+/−^ mice from the C57BL/6 *March5*^*tm1a*^ mice harbouring *Lox*P sites flanking *March5* exon 3 (Supplementary Fig. 1a,b). We verified that expression levels of MARCH5 protein and mRNA in three different tissues of the spleen, lung and liver were lower in *March5*^+/−^ mice than in *March5*^+/+^ mice (Supplementary Fig. 1c,d). When we intravenously challenged the *March5*^+/+^ and *Mach5*^+/−^ mice with replication-competent vesicular stomatitis virus (VSV, Indian strain), all *March5*^+/+^ mice died within 6 days of the infection, whereas 40% of *March5*^+/−^ mice still survived at 9 days (Fig. 1a), indicating that *March5*^+/−^ mice were more resistant to VSV infection. Analysis of VSV RNA levels in various organs (spleen, lung, liver and brain) from these mice revealed that VSV replication was significantly lower in *March5*^+/−^ mice than in *March5*^+/+^ mice, and remained lower for up to 5 days (Fig. 1b). We next investigated whether the low viral replication in *March5*^+/−^ mice was due to higher antiviral responses in these animals. *March5*^+/−^ mice produced higher levels of serum IFN-β and interleukin (IL)-6 than *March5*^+/+^ mice in response to infection with VSV-GFP (green fluorescent protein) virus or injection of poly(I:C) (Fig. 1c). We also observed higher expression of IFN-related genes in the organs of VSV-infected *March5*^+/−^ mice than in those of *March5*^+/+^ mice (Fig. 1d). These results strongly indicate that MARCH5 is involved in the innate response against RNA virus infection.

### MARCH5-deficient cells produce elevated IFN levels

**Figure 2:**
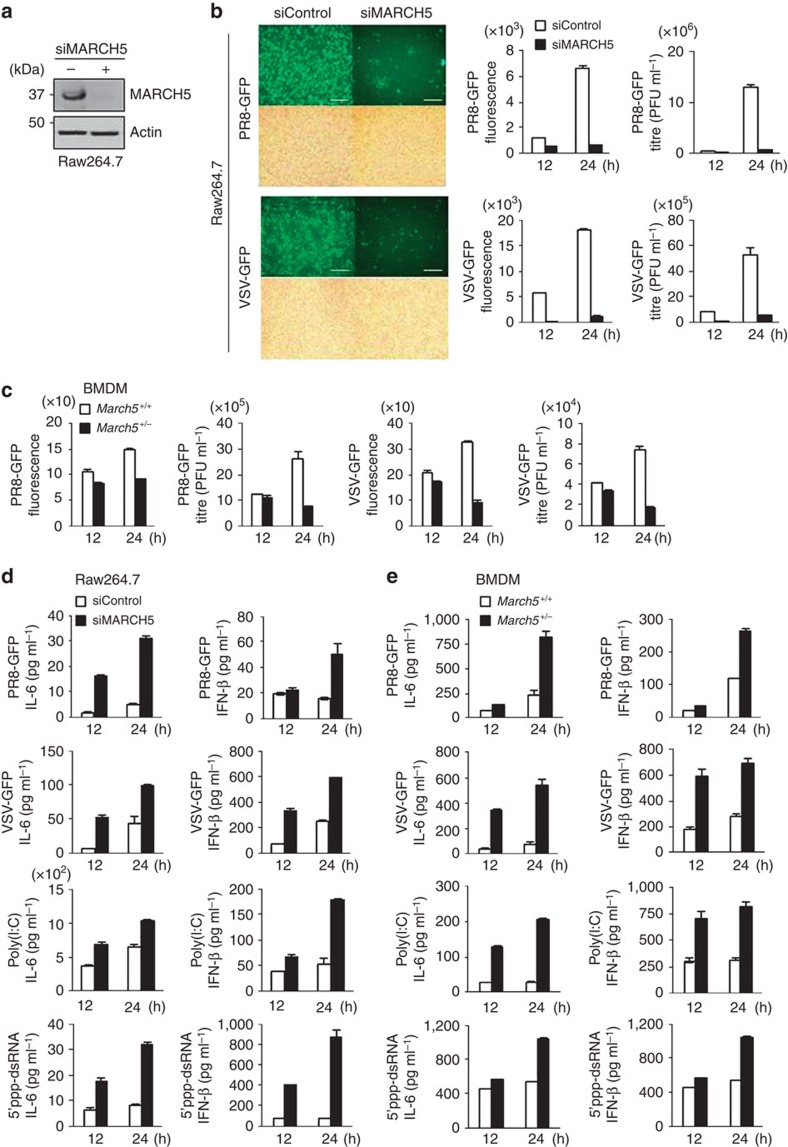
MARCH5-deficient immune cells produce elevated levels of type-I interferon and proinflammatory cytokines on infection with RNA viruses. (**a**) Immunoblot analysis of MARCH5 expression levels in MARCH5-depleted Raw264.7 cells. See full blots in Supplementary Fig. 8. (**b**) Twenty-four hours after infection, PR8-GFP or VSV-GFP virus replication assay by fluorescence microscopy (fluorescence, upper; phase-contrast microscopy, bottom) and virus titration by fluorescence analysis or plaque assay in siControl or siMARCH5 expressing Raw264.7 cells. Error bars, mean±s.e.m. (*n*=3). Scale bars, 100 μm. (**c**) Determination of the virus titration using fluorescence analysis or plaque assay in *March5*^+/+^ and *March5*^+/−^ BMDM cells infected with PR8-GFP or VSV-GFP virus. Error bars, mean±s.e.m. (*n*=3). (**d**,**e**) Bioassay of IFN-β or IL-6 (ELISA) in supernatants of MARCH5-depleted Raw264.7 (**d**) or *March5*^+/+^ and *March5*^+/−^ BMDM cells (**e**) infected or transfected with PR8-GFP, VSV-GFP, poly(I:C) or 5′ppp-dsRNA. After cells were transfected with siControl or siMARCH5 for 24 h, followed by infection with virus or transfection with poly(I:C) for indicated times. Error bars, mean±s.e.m. (*n*=3). All data are representative of at least three independent experiments.

Next, we investigated the modulatory effect of MARCH5 on innate immunity in various cell lines. A MARCH*5*-specific small interfering RNA (siRNA) efficiently inhibited the expression of endogenous MARCH5 in Raw264.7 macrophage-like cells (Fig. 2a). After siRNA-mediated knockdown (KO) of MARCH5, we infected Raw264.7 and HEK293T cells with VSV-fused GFP or PR8 (influenza virus)-fused GFP, and then analysed GFP expression and virus titre in these cells. Notably, fluorescence intensities of GFP and virus titre were significantly lower at 24 h after viral infection in cells transfected with MARCH5 siRNA than in control cells transfected with scrambled siRNA (Fig. 2b and Supplementary Fig. 2a). These findings were verified in bone marrow-derived macrophages (BMDMs) obtained from *March5*^+/+^ and *March5*^+/−^ mice. Viral replication of VSV and PR8 and GFP fluorescence intensity were also consistently reduced in BMDMs obtained from *March5*^+/−^ mice relative to those from *March5*^+/+^ mice (Fig. 2c). In consistent with our finding *in vivo* (Fig. 1), virus amplification in MARCH5-deficient cells was due to elevated production of IFN-β and the proinflammatory cytokine IL-6. Next, we applied four different stimulants of RLR signalling to Raw264.7 (Fig. 2d), BMDMs (Fig. 2e) and HEK293T cells (Supplementary Fig. 2b). Viral infection of VSV-GFP or PR8-GFP and transfection of poly(I:C) or 5′ppp-dsRNA, a synthetic ligand for RIG-I, increased the secretion of IFN-β and IL-6 from MARCH5-deficient cells. By contrast, infection of BMDMs from *March5*^+/+^ and *March5*^+/−^ mice with the DNA virus HSV or treatment with poly(dA:dT) had no significant effect on the secretion of either cytokine (Supplementary Fig. 2c). Finally, we confirmed that MAVS-dependent induction of IFN-β and IL-6 genes, as well as IFN-stimulated genes, was significantly elevated in MARCH5 knockout (KO) HEK293T cells after challenge with the VSV-GFP virus or 5′ppp-dsRNA (Supplementary Fig. 3). Taken together, the data strongly indicate that MARCH5 KO significantly exacerbates the type-I IFN response; thus, MARCH5 is a negative regulator of RLR signalling in innate immunity.

### The E3 ligase activity of MARCH5 is necessary

**Figure 3:**
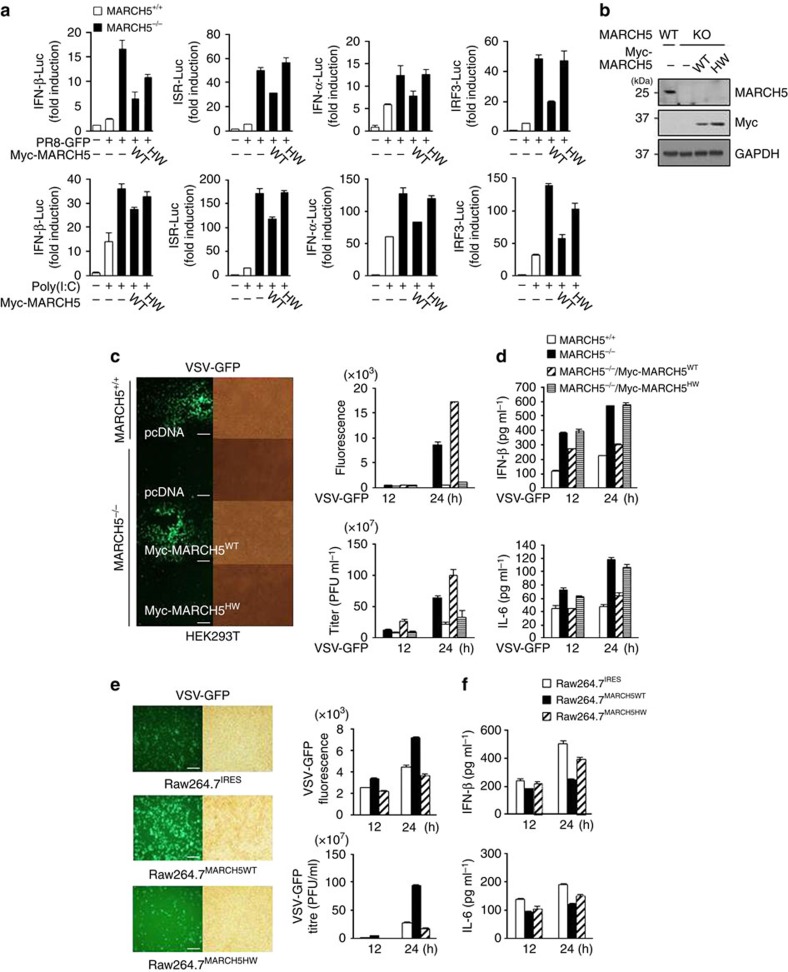
The E3 ligase activity of MARCH5 is necessary for inhibition of RLR-mediated antiviral signalling. (**a**) After Myc-MARCH5^WT^ or Myc-MARCH5^H43W^ expression vectors were introduced into MARCH5^−/−^ HEK293T cells, cells were infected with PR8-GFP or transfected with poly(I:C) for 24 h. Promoter activity of IFN-β, ISRE, IFN-α or IRF3. Graphs represent fold-induction relative to the luciferase activity in control cells. Error bars, mean±s.e.m. (*n*=3). (**b**) Immunoblot analysis of endogenous or ectopic expression levels of MARCH5 in MARCH5^+/+^ or MARCH5^−/−^ HEK293T cells. See full blots in Supplementary Fig. 8. (**c**,**d**) Twenty-four hours after infection, VSV-GFP replication assay by fluorescence microscopy analysis (**c**). Bioassay of IFN-β or IL-6 production (ELISA) (**d**). Error bars, mean±s.e.m. (*n*=3). Scale bars, 100 μm. (**e**,**f**) IRES, MARCH5^WT^-Flag or MARCH5^H43W^-Flag stably expressing Raw264.7 cells were stimulated by infection with VSV-GFP for 24 h. Twenty-four hours after infection, VSV-GFP replication assay by fluorescence microscopy analysis (left). Quantification of GFP intensity or virus titration by plaque assay (right; **e**). Bioassay of IFN-β or IL-6 production (ELISA) in supernatants of cultured Raw264.7 stable cells (**f**). Error bars, mean±s.e.m. (*n*=3). All data are representative of at least three independent experiments. Scale bars, 100 μm.

Because MARCH5 is a mitochondria-resident E3 ligase, we next addressed whether its E3 ligase activity is required for its ability to inhibit RLR signalling. A previous study predicted that histidine 43 in the RING domain of MARCH5 serves an ubiquitin-transfer function; consequently, the MARCH5^H43W^ mutant, in which His43 is replaced with tryptophan, lacks ubiquitin E3 ligase activity^10^. To determine whether this activity is required for inhibition of signalling, we generated a MARCH5 KO HEK293T cell line, re-expressed MARCH5^WT^ or MARCH5^H43W^ and used luciferase assays to determine the activities of the IFN-β, interferon-sensitive response element (ISRE), IFN-α and IRF3 promoters, which respond to the activation of the RLR signalling. As expected, the activities of these promoters were all higher in MARCH5^−/−^ HEK293T cells than in MARCH5^+/+^ cells on PR8-GFP infection or poly(I:C) treatment (Fig. 3a). The elevated activation of these promoters in MARCH5^−/−^ cells was inhibited by expression of MARCH5^WT^, but not MARCH5^H43W^. Expression levels of the corresponding proteins were confirmed using immunoblotting (Fig. 3b). Accordingly, viral VSV-GFP replication activity lowered in MARCH5^−/−^ HEK293T cells was enhanced by re-expression of MARCH5^WT^, but not by MARCH5^H43W^ (Fig. 3c). Consistent with the findings in Fig. 3a, higher secretion of IFN-β and IL-6 in MARCH5^−/−^ cells was suppressed by MARCH5^WT^, but not by MARCH5^H43W^ (Fig. 3d), indicating that the E3 ligase activity of MARCH5 is necessary for inhibition of RLR signalling. These findings were verified in Raw264.7 cell lines stably expressing MARCH5^WT^ or MARCH5^H43W^. Viral replication of VSV-GFP was enhanced in MARCH5^WT^-overexpressing Raw264.7 cells, but remained unchanged in MARCH5^H43W^-expressing cells (Fig. 3e). Similarly, secretion of IFN-β and IL-6 was lower in cells expressing MARCH5^WT^, but not in those expressing MARCH5^H43W^ (Fig. 3f). We also addressed the effects of MARCH5 on signalling downstream of MAVS by monitoring nuclear translocation of IRF3 on MAVS activation. To this end, we transfected normal HEK293T cells with MARCH5^WT^ or MARCH5^H43W^ together with GFP-IRF3 plasmids, and then stimulated the transfectants by infecting them with H1N1 influenza virus. A significant accumulation of GFP-IRF3 was evident 16 h after viral infection (Supplementary Fig. 4a). Overexpression of MARCH5^WT^ significantly decreased the amount of nuclear IRF3, whereas MARCH5^H43W^ had little effect on IRF3. Finally, we used reverse transcriptase–PCR (RT–PCR) to determine the expression level of IFN-β- and IFN-related genes after the poly(I:C) treatment. MARCH5^WT^ suppressed expression of these genes; this effect was most prominent 12 h after stimulation. By contrast, introduction of MARCH5^H43W^ into these cells had no effect (Supplementary Fig. 4b). Together, the data suggest that the E3 ubiquitin ligase function of MARCH5 is necessary for inhibition of RLR-mediated antiviral signalling.

### MARCH5 suppresses the MAVS-mediated type-I IFN signalling

**Figure 4:**
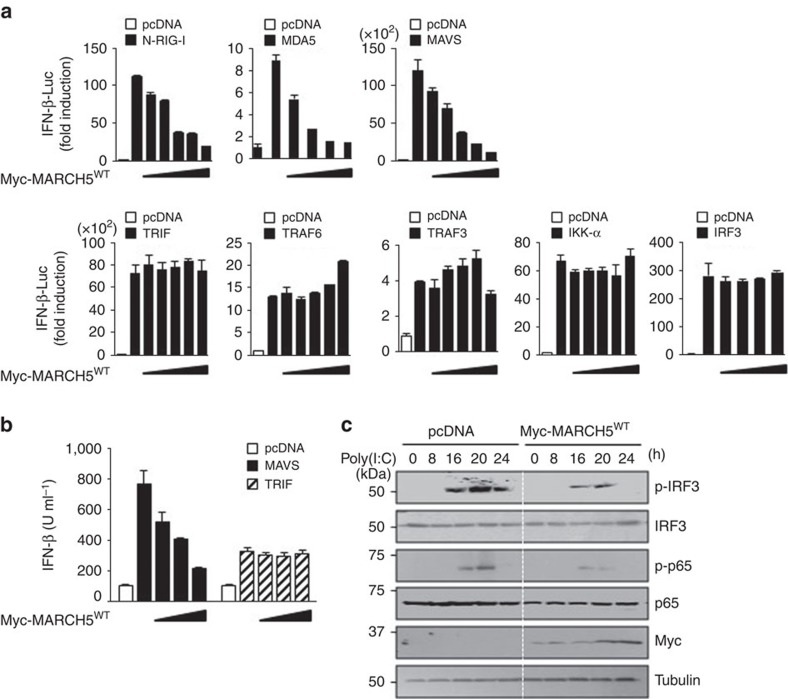
MARCH5 suppresses the MAVS-mediated type-I IFN signalling. (**a**) IFN-β promoter activity in N-RIG-I, MDA5, MAVS, TRIF, TRAF-6, TRAF-3, IKK-α or IRF3 with Myc-MARCH5^WT^ (0, 50, 100, 200, 400 and 800 ng)-transfected HEK293T cells for 36 h. Graphs represent fold-induction of IFN-β relative to the luciferase activity in control cells. Error bars, mean±s.e.m. (*n*=3). (**b**) Bioassay of IFN-β (ELISA) in supernatants of HEK293T cells transfected with MAVS or TRIF with Myc-MARCH5^WT^ (0, 100, 200 and 400 ng). Error bars, mean±s.e.m. (*n*=3). (**c**) Immunoblotting of p-IRF3, p-p65, total IRF and total p65 in lysates of HEK293T cells. After transfection with control or Myc-MARCH5^WT^ for 24 h, cells were stimulated by transfection with poly(I:C) for the indicated time. All data are representative of at least three independent experiments. See full blots in Supplementary Fig. 8.

Following viral infection, various components of the type-I IFN signalling pathway play a role in the subsequent cellular response. Therefore, we next attempted to determine which step in RLR signalling is the target of MARCH5. Binding of RIG-I/MDA5 to RNA ligands initiates signalling cascades by interacting with MAVS, leading to the activation of the TRAF-3/TBK1/IRF3 and TRAF-6/IKK-α,β,γ/NF-κB pathways. Because the type-I IFN signalling pathway can be also activated through Toll-like receptors (TLRs), we also tested whether TRIF and TRAF-3 are involved. To this end, we co-transfected cells with the IFN-β-Luc reporter plasmid and expression vectors encoding N-RIG-I, MDA5, MAVS, the Toll-like receptor adapter TRIF, TRAF-6, TRAF-3, IKK-α or IRF3 along with increasing amounts of a MARCH5 expression plasmid. MARCH5 inhibited N-RIG-I-, MDA5- or MAVS-induced IFN-β-Luc activity in a dose-dependent manner, whereas activation of the IFN-β promoter induced by TRIF was not affected (Fig. 4a). Moreover, the IFN-β-Luc activity induced by TRAF-6, TRAF-3, IKK-α or IRF3 was unaffected by MARCH5 overexpression, indicating that MARCH5 specifically inhibited RLR-mediated MAVS signalling in mitochondria. Likewise, MAVS-stimulated production of IFN-β was diminished by MARCH5 overexpression in a dose-dependent manner (Fig. 4b). By contrast, TRIF-mediated IFN-β production was not affected by MARCH5. The effects of MARCH5 on signalling downstream of MAVS were also verified in HEK293T cells after stimulation with poly(I:C). Transfected poly(I:C) robustly increased IRF3 and p65 phosphorylation in a time-dependent manner, whereas these effects were significantly attenuated in cells transfected with Myc-MARCH5^WT^ (Fig. 4c). These results demonstrate that MARCH5 specifically targets MAVS-mediated type-I IFN signalling. We verified that interferon-stimulated gene (ISG) induction after IFN-β treatment was not affected by MARCH5 (Supplementary Fig. 4c).

### March5 directly binds to MAVS

**Figure 5:**
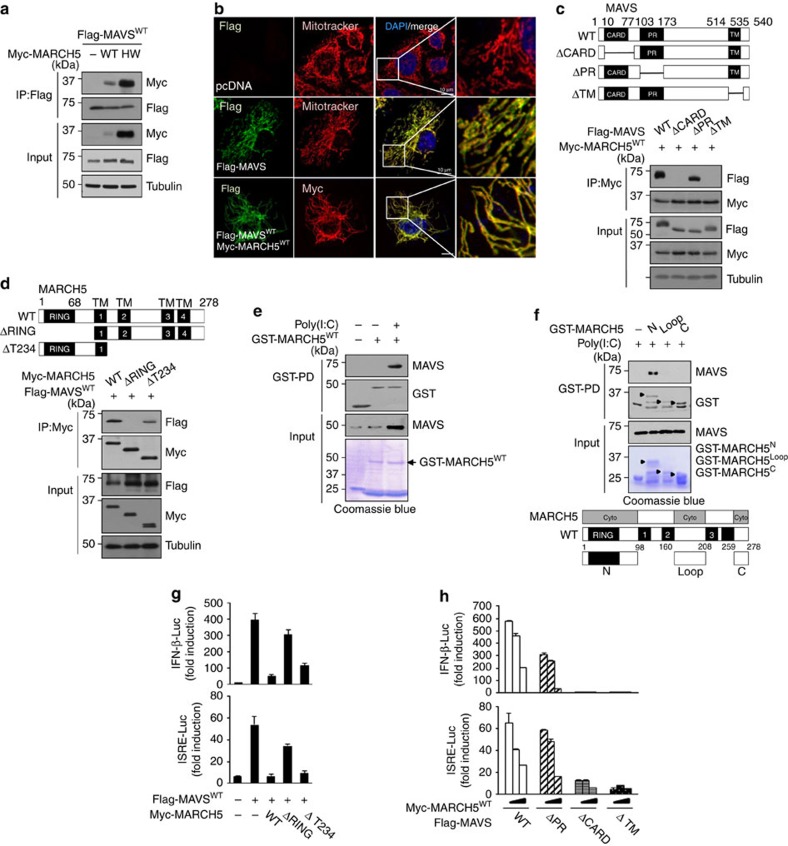
MARCH5 binding to MAVS is necessary for the inhibition of type-I IFN responses. (**a**) Immunoprecipitation between Myc-MARCH5 and Flag-MAVS in HEK293T cells. HEK293T cells were co-transfected with indicated plasmids for 36 h and interaction was analysed by immunoblotting with indicated antibody. (**b**) Confocal microscopy analysis of Flag-MAVS^WT^ and Myc-MARCH5^WT^ localization in HEK293T cells. Scale bars, 10 μm. (**c**,**d**) Immunoprecipitation analysis of Flag-MAVS or Myc-MARCH5-truncated mutants. HEK293T cells were co-transfected with indicated plasmids and interaction was analysed by immunoblotting with indicated antibodies. Flag-MAVS fragments (WT: full length, ΔCARD; Δ10–77, ΔPR; Δ103–173, ΔTM; Δ514–535) (**c**). Myc-MARCH5 fragments (WT; full length, ΔR; 76–278, ΔT234; 1–139; **d**). (**e**,**f**) GST pull-down assays using GST-tagged wild-type and mutants of MARCH5. The GST-conjugated MARCH5 wild-type protein was purified from insect and incubated with cell lysates treated with/without poly(I:C) (**e**). The GST-MARCH5 mutants were purified from bacteria and their interactions to MAVS were analysed immunoblotting with anti-Flag antibody. The bottom panel shows the Coomassie Blue-stained gel (**f**). (**g**) IFN-β and ISRE promoter activity in Flag-MAVS expressing HEK293T cells transfected with Myc-MARCH5 fragments. Error bars, mean±s.e.m. (*n*=3). (**h**) Promoter activity of IFN-β and ISRE in HEK293T cells transfected with Myc-MARCH5^WT^ and Flag-MAVS fragment constructs. Graphs represent fold-induction relative to the luciferase activity in control cells. Error bars, mean±s.e.m. (*n*=3). All data are representative of at least three independent experiments. See full blots in Supplementary Fig. 8.

Next, we investigated whether inhibition of MAVS-mediated type-I IFN signalling is mediated by its binding of MARCH5 to MAVS. In co-immunoprecipitation experiments, both Myc-MARCH5^WT^ and Myc-MARCH5^H43W^ interacted with FLAG-tagged MAVS in HEK293T cells (Fig. 5a). These interactions appeared to occur in the mitochondria: immunofluorescence staining revealed colocalization of exogenously expressed FLAG-MAVS^WT^ and Myc-MARCH5^WT^, especially at mitochondrial clusters (Fig. 5b). To identify the domains responsible for the interaction between MARCH5 and MAVS, we generated a series of deletion mutants of MARCH5 and MAVS, and then carried out co-immunoprecipitation analyses using these constructs. Both the caspase activation and recruitment domain (CARD) and tramsmembrane (TM) domain of MAVS were required for its interaction with MARCH5 (Fig. 5c), and deletion of the RING domain of MARCH5 significantly reduced MARCH5 binding to MAVS (Fig. 5d). In keeping with these findings, the expression levels of MAVS^WT^ and MAVS^ΔPR^ were reduced by MARCH5^WT^, whereas MAVS^ΔCARD^ and MAVS^ΔTM^ levels were not affected by MARCH5^WT^ (Supplementary Fig. 5a). Likewise, FLAG-MAVS^WT^ levels were not affected by overexpression of MARCH5^ΔRING^ but were suppressed by MARCH5^WT^ and MARCH5^ΔT234^ (Supplementary Fig. 5b). Importance of the RING domain in MARCH5 and MAVS interactions was verified by glutathione *S*-transferase (GST)-pull-down assay. The GST-conjugated MARCH5 wild-type and mutant proteins^35^ were purified from insect (Fig. 5e) and bacteria lysates (Fig. 5f), respectively, and incubated with cell lysates treated with/without poly(I:C). We found that MARCH5 directly interacted with MAVS and the N terminus of MARCH5 containing the RING domain kept bound to MAVS, whereas the cytosolic loop of MARCH5 (AAs 160–208) and C terminus (AAs 259–278) lost their binding to MAVS (Fig. 5f). To address the functionality of this interaction, we carried out IFN-β and ISRE luciferase reporter assays in cells co-expressing wild-type or mutant MAVS and MARCH5 proteins. The MAVS-mediated increase in luciferase activity was significantly decreased by MARCH5^WT^ or MARCH5^ΔT234^, whereas luciferase activity remained high on co-expression of MARCH5^ΔRING^ (Fig. 5g). Together, these results suggest that the RING domain of MARCH5 is necessary for its binding to MAVS, as well as its inhibition of type-I IFN responses (Fig. 3a). In addition, the MARCH5^ΔT234^ mutant, which contains one TM domain, was still able to exert its inhibitory function. In the immunofluorescence staining shown in Supplementary Fig. 5c, we observed that MARCH5^ΔT234^ mutant proteins displayed a punctuated form that partly overlaid with mitochondria. Similarly, only the MAVS^WT^ and MAVS^ΔPR^ constructs were capable of activating IFN-β and ISRE luciferase reporters; these effects, in turn, were suppressed in a dose-dependent manner by co-expression of MARCH5 (Fig. 5h). The CARD domain of MAVS is necessary for the interaction with RIG-I and its subsequent oligomerization^36^,^37^. In the absence of the TM, MAVS hardly positioned to the mitochondria membrane. Thus, the data suggest that binding of the MARCH5 RING domain to the CARD domain of MAVS at the mitochondria is necessary for negative regulation of type-I IFN responses.

### MARCH5 reduced levels of the MAVS aggregates

**Figure 6:**
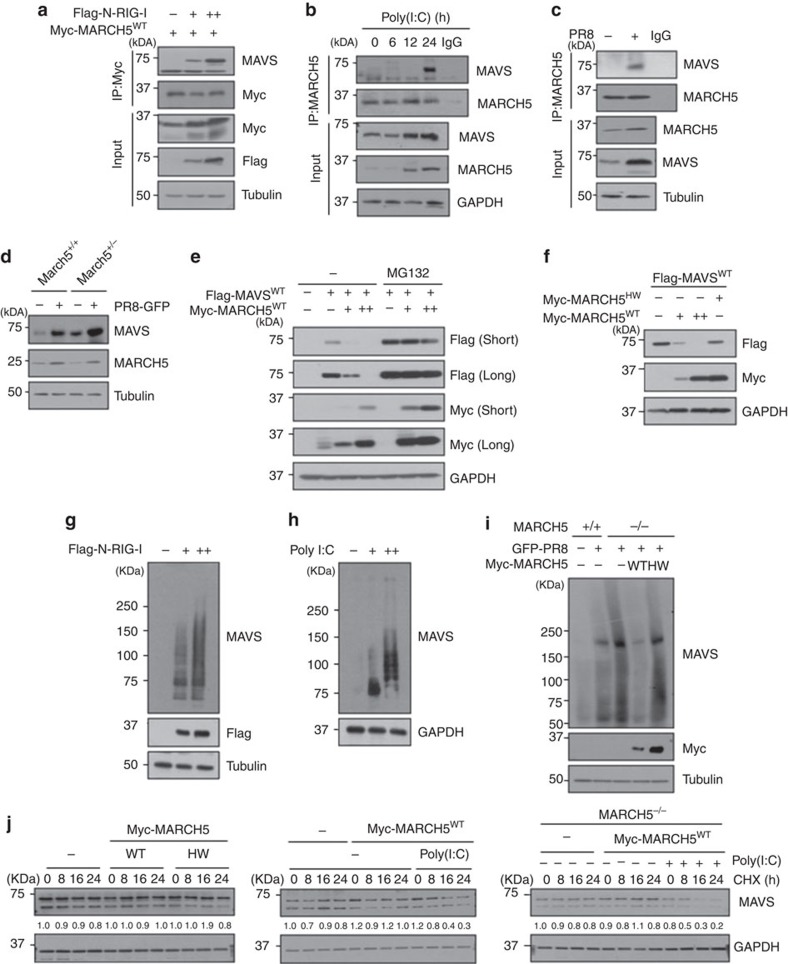
MARCH5 reduced the MAVS aggregates via the proteasome-mediated degradation pathway. (**a**,**b**) Interaction between MARCH5 and MAVS in HEK293T cells. Cells were transfected with Flag-N-RIG-I (**a**) or poly(I:C) (**b**); interaction was determined by immunoblotting with anti-MAVS antibody after lysates were immunoprecipitated with anti-MARCH5 or anti-Myc antibody. (**c**) After cells were infected with PR8 virus for 24 h, cell lysates were immunoprecipitated with anti-MARCH5 antibody or control IgG. Interaction was evaluated using anti-MAVS antibody. (**d**) Immunoblot analysis of endogenous MAVS expression levels in *March5*^+/+^ and *March5*^+/−^ BMDM cells infected with PR8-GFP. (**e**) Immunoblot analysis of Flag-MAVS expression levels in HEK293T cells. Cells were transfected with Flag-MAVS^WT^ and Myc-MARCH5^WT^, with or without treatment of MG132 (10 μM). (**f**) Immunoblot analysis of Flag-MAVS^WT^ expression levels in HEK293T cells. Cells were co-transfected with Flag-MAVS and Myc-MARCH5^WT^ or Myc-MARCH5^H43W^. (**g**,**h**) Cells were transfected with Flag-N-RIG-I (**g**) or poly(I:C) (**h**) for 36 h. Lysates were mixed with or without 6 × native sample buffer, followed by SDS–PAGE. (**i**) After Myc-MARCH5^WT^ or Myc-MARCH5^H43W^ expression vector was reintroduced into MARCH5^−/−^ HEK293T cells, cells were infected with PR8-GFP. The lysates were mixed with or without 6 × native sample buffer, followed by SDS–PAGE. (**j**) CHX chase analysis of endogenous MAVS in MARCH5^+/+^ or MARCH5^−/−^ HEK293T cells. After Myc-MARCH5^WT^-expressing vector was reintroduced to MARCH5^−/−^ HEK293T cells, cells were exposed to CHX for indicated times before harvest, with or without transfection with poly(I:C). Numbers below lanes indicate MAVS expression levels relative to GAPDH. All data are representative of at least three independent experiments. See full blots in Supplementary Fig. 8.

MAVS is strategically localized to mitochondria, where it is necessary for RIG-I signalling. Because MARCH5 also localizes to the mitochondrial outer membrane, we investigated when the interaction of MARCH5 with MAVS occurred. Co-immunoprecipitation revealed that Myc-MARCH5 overexpressed in HEK293T bound endogenous MAVS only when RIG-I was activated by overexpression of N-RIG-I (Fig. 6a). Similarly, endogenous MARCH5 bound to MAVS only after poly(I:C) transfection (Fig. 6b) or PR8 infection (Fig. 6c). Notably, binding of MARCH5 to MAVS occurred 24 h after the poly(I:C) treatment. Thus, the MARCH5 binding to MAVS was induced only under the RIG-I stimulation. Likewise, we observed higher MAVS accumulation in *March5*^*+/−*^ mice than in *March*^*+/+*^ mice on PR8-GFP infection (Fig. 6d). Next, we addressed whether the MARCH5 binding of MAVS promoted proteasomal degradation of MAVS. We found that exogenously expressed MARCH5^WT^ induced a dose-dependent decrease in FLAG-MAVS^WT^ protein levels in HEK293T cells; this reduction was absent in the presence of the proteasome inhibitor MG132 (Fig. 6e). By contrast, introduction of MARCH5^H43W^ did not reduce the level of the MAVS protein (Fig. 6f), suggesting that MARCH5 E3 ligase promotes MAVS degradation through a proteasome-dependent pathway.

Under RIG-I stimulation, MAVS forms prion-like aggregates that potently propagate downstream signalling^21^,^22^,^23^. MARCH5 bound MAVS only under conditions of RIG-I stimulation, in which MAVS forms aggregates^21^. In semi-denaturing detergent polyacrylamide gel electrophoresis, we also observed a smear of SDS-resistant high-molecular-weight MAVS aggregates 24 h after N-RIG-I or poly(I:C) transfection (Fig. 6g,h). Likewise, high-molecular-weight MAVS aggregates were induced by viral infection of PR8 at 24 h. Importantly, formation of the MAVS aggregates induced by viral infection was intensified in MARCH5 KO cells; re-expression of MARCH5^WT^, but not MARCH5^H43W^, significantly reduced the levels of aggregates (Fig. 6i). Likewise, the MAVS aggregates stimulated by N-RIG-I were significantly reduced by overexpression of MARCH5 in a dose-dependent manner (Supplementary Fig. 6). These results suggest that MARCH5 does not interact with monomeric MAVS at the mitochondrial membrane. Under RIG-I stimulation, MARCH5 recognizes MAVS aggregates and targets them for proteasome-mediated degradation. We also introduced MARCH5^WT^ or MARCH5^H43W^ expression plasmids into HEK293T cells and monitored the degradation patterns of endogenous MAVS protein in the presence of the protein translation inhibitor cycloheximide (CHX). At 24 h after CHX treatment, MAVS levels were slightly reduced (80–90% of initial levels), and no significant differences in MAVS levels were found among cells into which pcDNA, MARCH5^WT^ or MARCH5^H43W^ had been introduced (Fig. 6j, left panel). Notably, however, the poly(I:C) treatment accelerated degradation of MAVS protein (Fig. 6j, middle panel), reducing MAVS protein to ∼25% of the initial level at 24 h. These findings are consistent with the observation that re-expression of MARCH5^WT^ in MARCH5^−/−^ HEK293T cells triggered MAVS degradation only in the context of poly(I:C) stimulation (Fig. 6j, right panel). Together, these data suggest that MARCH5 reduces the level of MAVS aggregates through the proteasome-mediated degradation pathway. To our knowledge, mitochondria-resident MARCH5 is the first negative regulator shown to target MAVS aggregates.

### MARCH5 transfers ubiquitin to Lys7 and Lys500 of MAVS

**Figure 7:**
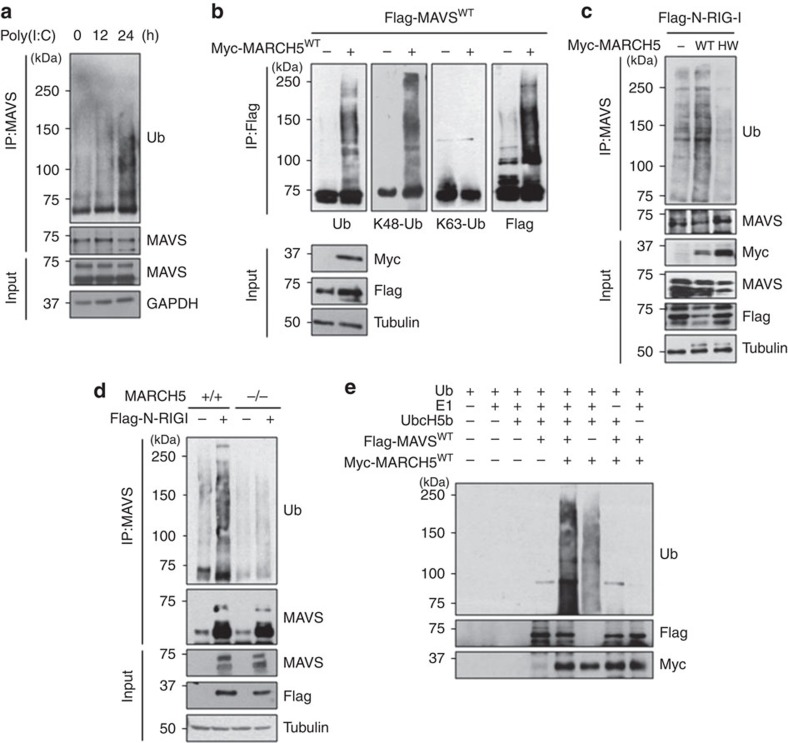
MARCH5 transfers ubiquitin to MAVS through a K48 linkage. (**a**) Ubiquitination assay of endogenous MAVS by using anti-MAVS antibody in poly(I:C)-transfected HEK293T cells. Levels of ubiquitination were determined by anti-Ub antibody. (**b**,**c**) Ubiquitination assays in Flag-MAVS^WT^ and Myc-MARCH5^WT^-expressing HEK293T cells. After cells were transfected with Flag-MAVS^WT^ and control or Myc-MARCH5^WT^, ubiquitination levels of Flag-MAVS were determined by immunoblotting with anti-Ub, K48-Ub or K63-Ub-specific antibodies (**b**). Cells were co-transfected with Flag-N-RIG-I and Myc-MARCH5WT or H43W. Endogenous ubiquitination levels of MAVS were determined by anti-Ub antibody after IP with endogenous anti-MAVS antibody (**c**). (**d**) Ubiquitination of endogenous MAVS in MARCH5^+/+^ or MARCH5^−/−^ HEK293T cells transfected with Flag-N-RIG-I. (**e**) *In vitro* ubiquitination assay of Flag-MAVS. Myc-MARCH5^WT^ and Flag-MAVS^WT^ were incubated with a reaction mixture with ubiquitin, E1, UbcH5b as E2, and ubiquitination levels were determined by immunoblotting with anti-Ub antibody. See full blots in Supplementary Fig. 8.

Next, we characterized the patterns of MARCH5-mediated ubiquitination of MAVS. The poly(I:C) transfection into HEK293T cells significantly increased MAVS polyubiquitin complexes at 24 h (Fig. 7a). Notably, the polyubiquitin chains in MARCH^WT^-expressing cells contained lysine (K) 48-linked ubiquitin, but not K63-linked ubiquitin (Fig. 7b). MARCH5-induced ubiquitination was not observed in MARCH5^H43W^-expressing cells (Fig. 7c). Moreover, challenging cells by overexpressing N-RIG-I increased MAVS ubiquitination in MARCH5^+/+^ HEK293T cells, but not in MARCH5^−/−^ HEK293T cells (Fig. 7d). We also noticed that the levels of endogenous MAVS were increased after N-RIG-I transfection, probably because MAVS aggregates are known to be resistant to proteases^21^. Finally, *in vitro* ubiquitination assays verified that MARCH5, in combination with an E1 ubiquitin-activating enzyme and the E2 ubiquitin-conjugating enzyme UbcH5b, effectively transferred ubiquitin to MAVS (Fig. 7e). In the absence of MARCH5, no ubiquitination occurred, and in the absence of MAVS, MARCH5 was autoubiquitinated. Thus, the data indicate that MARCH5 transfers the K48-linked ubiquitin to MAVS under RIG-I stimulation.

**Figure 8:**
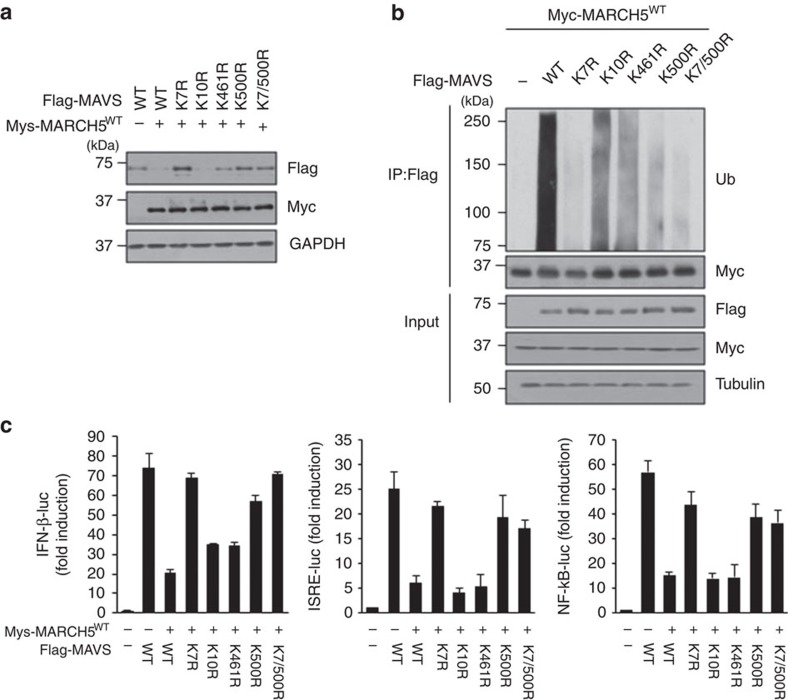
MARCH5 transfers ubiquitin to Lys7 and Lys500 residues of MAVS. (**a**) Expression of Flag-MAVS lysine mutants by Myc-MARCH5^WT^ in HEK293T cells. (**b**) Ubiquitination assay of Flag-MAVS lysine mutants by Myc-MARCH5^WT^. Ubiquitination of MAVS lysine mutants was assessed by immunoblotting with anti-Ub antibody. (**c**) Promoter assay of IFN-β, ISRE or NF-κB in HEK293T cells transfected with Myc-MARCH5^WT^ and Flag-MAVS^WT^ or lysine mutants for 36 h. Graphs represent fold-induction relative to the luciferase activity in control cells. Error bars, mean±s.e.m. (*n*=3). All data are representative of at least three independent experiments. See full blots in Supplementary Fig. 8.

Next, to identify the lysine (K) residues of MAVS to which ubiquitin is attached, we first performed an *in silico* search for ubiquitin-binding sites on MAVS using the UbPred programme and PhosphoSitePlus (http://www.phosphosite.org). This analysis revealed four putative lysine target residues: K7, K10, K461 and K500. We then generated FLAG-MAVS mutants in which each of these lysine residues was replaced with arginine (R). The steady-state levels of these point-substitution mutant proteins were comparable; however, they responded differently to MARCH5. MAVS^K10R^ was as sensitive to MARCH5 as MAVS^WT^, whereas neither MAVS^K7R^ nor MAVS^K500R^ was affected by MARCH5^WT^ overexpression (Fig. 8a). MAVS^K461R^ protein levels were decreased by MARCH5^WT^, but to a lesser extent than MAVS^K10R^ or MAVS^K500R^. *In vivo* ubiquitination assays verified a substantial decrease in ubiquitin conjugation in the MAVS^K7R^ and MAVS^K500R^ mutants (Fig. 8b). To address the functional importance of ubiquitination on these Lys sites in the type-I IFN responses, HEK293T cells were transfected with these ubiquitin conjugation-defective MAVS mutants and Myc-MARCH5^WT^ together with IFN-β, ISRE or NF-κB luciferase reporter constructs, MAVS^K7R^ and MAVS^K500R^ fully retained the ability to increase luciferase activity from all promoters tested, even in the presence of MARCH5^WT^ (Fig. 8c). However, MAVS^WT^ as well as the MAVS^K10R^ and MAVS^K461R^ mutants only weakly activated these promoters when co-expressed with MARCH5^WT^. Taken together, these data demonstrate that MARCH5 transfers ubiquitin to Lys7 and Lys500 of MAVS through a K48 linkage and accelerates MAVS degradation.

## Discussion

On the mitochondrial membrane, MAVS acts as a critical adaptor protein of the RLR signalling pathway that links upstream viral RNA recognition to downstream signal activation to control viral replication^17^,^18^,^19^. A remarkable feature of MAVS is the formation of prion-like aggregates that are not only functional but also potently propagate downstream signalling^21^,^22^,^23^. The persistence of MAVS signalling, however, results in an excessive immune response, leading to detrimental effects on the host. Understanding the mechanism by which MAVS aggregation is initiated, and how it is subsequently resolved, is crucial for a complete understanding of innate immunity.

In this study, we provided several lines of evidence that MARCH5 is a potent negative regulator of MAVS-mediated antiviral innate immunity, and that it is a unique mitochondria-resident E3 ubiquitin ligase. First, *March5*^+/−^ mice exhibited reduced morbidity on VSV infection because of low viral replication. Second, knockout of endogenous MARCH5 in immune cells resulted in reduced viral replication that correlated well with high type-I IFN responses. Third, MARCH5 bound MAVS at the mitochondrial membrane only under conditions of RIG-I activation. Fourth, MARCH5 could reduce the level of MAVS aggregates via proteasome-mediated degradation. Thus, MARCH5 is the crucial mitochondria-resident regulator that coordinates the MAVS signalling via timely degradation of the aggregated MAVS. This MARCH5-mediated quality-control provides an efficient way to prevent excessive host immunity.

The MARCH proteins were initially identified as a family of viral ubiquitin ligases^2^,^38^,^39^,^40^; to date, 11 mammalian homologues have been identified. Both viral E3 ligases and mammalian homologues have been proposed to act as immune modulators in viral immune evasion strategies^40^,^41^,^42^,^43^. Most mammalian MARCH proteins contain two or more transmembrane domains that target them to intracellular organelles or the plasma membrane. So far, the reported functions of mammalian MARCH proteins have been limited to downregulation of immunomodulatory surface proteins of immune cells. MARCH4 and MARCH9 decrease the surface expression of major histocompatibility complex class 1 (MHC 1) and/or Intercellular Adhesion Molecule 1 (ICAM1) in T and B cells^44^. In addition, MARCH8 triggers degradation of IL-1 receptor accessory protein^45^. MARCH5 is the first MARCH family member shown to be involved in TLR7 signalling. MARCH5 catalyses polyubiquitination of TANK (TRAF family member-associated NF-κB activator) through a K63-Ub linkage that leads to activation of NF-κB (ref. 46). In this study, we found that MARCH5 facilitated the degradation of MAVS after RIG-I mediated recognition of cytosolic viral dsRNAs, thereby controlling excessive production of type-I interferon. To our knowledge, this is the first demonstration of a negative modulatory function for a MARCH family protein in antiviral innate immunity. Unlike other cytosolic E3 ligases that negatively regulate RLR signalling pathways, MARCH5 is a mitochondrial protein that directly binds and degrades activated MAVS oligomers following viral infection. AIP4 E3 ligase-mediated degradation of MAVS requires a scaffolding protein, PCBP2 (ref. 28). Similarly, Ndfip1 leads to the recruitment of Smurf1 E3 ligase, triggering proteasome-mediated degradation of MAVS^31^. Other E3 ligases, such as cytosolic TRIM25 (ref. 47) and RNF5 in the endoplasmic reticulum^48^, have also been shown to accelerate MAVS degradation after viral infection.

In non-activated cells, MAVS is anchored to the mitochondrial outer membrane in its monomeric form^21^,^22^,^49^,^50^. During viral infection, RIG-I and MAVS proteins undergo conformational changes that are essential for antiviral signalling^51^. Of particular note, MAVS forms well-ordered, functional aggregates, and these MAVS fibrils behave like prions^21^. In this study, we showed that MARCH5 did not interact with MAVS in unstimulated cells, and that its binding to MAVS was induced by stimulation with virus, N-RIG-I or poly(I:C) treatment (Fig. 6a–c). MARCH5 binding to MAVS occurred 12–24 h after viral infection or mimetic stimulation (Fig. 6a–c). It is of note that MARCH5 protein is upregulated after virus infection or mimetic stimulation (Fig. 6a–c). In fact, a previous study reported that MARCH5 controls its own protein level through autoubiquitination^12^. Likewise, analysis of the ubiquitination pattern of MARCH5 revealed that this protein is highly ubiquitinated in the absence of N-RIG-I stimulation (Supplementary Fig. 7); consequently, it is maintained at a low steady-state level. On N-RIG-I stimulation, its autoubiquitination activity was significantly reduced, leading to accumulation of MARCH5 protein. Thus, MARCH5 levels are upregulated following virus infection or mimetic stimulation, and these characteristics of MARCH5 enable selective and timely interaction to the activated MAVS oligomer. These results strongly suggest that MARCH5 is likely to bind only to the activated MAVS oligomers, but not to the non-activated monomeric MAVS. In fact, we observed that the level of high-molecular-weight MAVS aggregates induced by influenza virus infection was significantly reduced by MARCH5 (Fig. 6i). At present, it remains unclear how MARCH5 distinguishes the monomeric MAVS from the MAVS oligomer. However, previous work showed that MARCH5 selectively facilitates the degradation of protein aggregates and misfolded superoxide dismutase 1 accumulated at mitochondria^14^,^15^. Thus, MARCH5 plays an important role in protein quality control and maintenance of mitochondrial homeostasis. Our deletion analysis showed that the N-terminal CARD domain of MAVS was necessary for MARCH5 binding (Fig. 5c). Because the CARD domain is required for MAVS polymerization and RIG-I-dependent IRF3 activation^52^, the inability of the MAVS^ΔCARD^ mutant to bind MARCH5 may be due to a failure of MAVS polymerization. Furthermore, we identified amino acids Lys7 and Lys500 of MAVS as critical for MARCH5-mediated ubiquitination (Fig. 8b). Paz *et al*.^53^ reported that K63-linked ubiquitination at Lys500 of MAVS leads to recruitment of inhibitor of κB kinase ɛ. In this study, we demonstrated that Lys500 residue of MAVS is also a target for K48-linked ubiquitination by MARCH5.

In summary, we identified the E3 ubiquitin ligase MARCH5 as a novel mitochondrial regulator of the immune response against RNA virus infection. MARCH5 targets the activated MAVS oligomer and attenuates MAVS-mediated IFN signalling through K48-linked ubiquitination and degradation of MAVS. Thus, MARCH5 is a crucial mitochondria-resident regulator that coordinates the MAVS signalling via timely degradation of activated MAVS oligomers, and this MARCH5-mediated quality control provides an efficient way of preventing excessive host immunity. Improved understanding of MARCH protein-mediated modulation of RLR signalling may facilitate development of new strategies for targeting the RLR pathway to achieve therapeutic control of viral infection and enhancement of the innate immune response.

## Methods

### Viruses and plasmids

Recombinant VSV-GFP^54^ and PR8-GFP viruses^55^ were kindly provided by Dr Sean P. Whelan (Harvard Medical School, Boston, MA, USA) and Dr Adolfo García-Sastre (Mount Sinai School of Medicine, New York, NY, USA), respectively. Viral stocks were prepared and kept at −70 °C before use. Myc-tagged MARCH5-truncated mutants (ΔRING, ΔT234) were generated using Myc-MARCH WT as a template, described in ref. 16. To generate pIRES-MARCH5-Flag, MARCH5 genes were amplified from template DNA using PCR and cloned into plasmid pEF-Flag-IRES-puro using AflII and XbaI for selection of stable transfectants. Flag-MAVS WT, ΔCARD, ΔPR and ΔTM constructs were kindly provided by Dr Zhijian J. Chen^24^. Flag-RIG-I and Flag N-RIG-I were kindly provided by Dr Hui Zhong^29^. Flag-MAVS lysine mutants (K7R, K10R, K461R and K500R) were generated with PCR, using a site-directed mutagenesis kit (Intron). Reporter gene constructs^47^ were provided by Dr Jae U. Jung (University of Southern California, Los Angeles, CA, USA).

### Mice and *in vivo* viral infection

C57BL/6 *March5*^*tm1a*^ mice harbouring *Lox*P sites flanking *March5* exon 3 were purchased from the European Mouse Mutant Archive and placed in the same genetic background to generate heterozygous *March*5 mice. All mice were maintained in a specific pathogen-free animal facility. Offspring were genotyped using PCR with specific primer. The following primer set was used: forward 5′- GTGACACTACTTTTGATGTGAAG -3′, reverse 5′- ATGCTACAGCTCATGTGTAAG -3′ For mice viral infection experiments, 5–6-week-old male mice were infected with VSV Indiana virus (2 × 10^8^ p.f.u. per mouse) or 200 mg of poly(I:C) via tail intravenous injection. All challenge tests were conducted inside an approved BSL-3+ facility under appropriate conditions.

### Cell culture and transfections

HEK293T (ATCC, ACS-4500), Raw264.7 (ATCC, TIB-71) cells were cultured in DMEM (Invitrogen) supplemented with 10% heat-inactivated fetal bovine serum (FBS) and 1% penicillin–streptomycin (GIBCO BRL) in a 5% CO_2_ incubator at 37 °C. THP-1 cells were cultured in RPMI supplemented with 10% heat-inactivated FBS and 1% penicillin–streptomycin (GIBCO BRL). BMDMs were isolated from tibias and femurs of 5–6-week-old male mice. After collection of bone marrow, red blood cells were lysed with ACK lysis buffer (GIBCO BRL) and pellet cells were cultured in 10% L929-cell conditioned medium containing granulocyte–macrophage colony-stimulating factor at 37 °C in a 5% CO_2_ incubator. Plasmid DNA constructs were transfected using polyethylenimine (Polysciences). Poly(I:C) (InvivoGen), siRNA or 5′ppp-dsRNA (InvivoGen) was transfected using Lipofectamine 2000 (Invitrogen) according to the manufacturer's instructions. Raw264.7 cells stably expressing pIRES, pIRES-MARCH5^WT^-Flag or pIRES-MARCH5^HW^-Flag were grown in 1-μg ml^−1^ puromycin containing DMEM after selection with 2 μg ml^−1^ of puromycin for 2 weeks.

### Generation of MARCH5 KO cells

To generate HEK293T MARCH5 KO cells, TALEN (transcription activator-like effector nuclease) plasmids targeting exon 2 of MARCH5 were engineered by ToolGen Inc. (Korea)^56^. The MARCH5-specific TALEN plasmid contains the sequences of 5′- TGATGAAGATGATAGAACAG -3′ (TALEN-L) and 5′- TCCTCTGCACCTGCATGGTC -3′ (TALEN-R) that are linked by the EN target sequence. To enrich the MARCH5 KO cells, the pRG2S surrogate reporter plasmids^56^ were co-transfected into HEK293T cells using Lipofectamine according to the manufacturer's protocol. The pRG2S reporters are composed of genes encoding two fluorescent proteins (red and green) and the frameshift mutations by endonuclease (EN) lead to restoration of the green fluorescent protein gene. At 48 h post transfection, the cells expressing green fluorescence were sorted under FACS (FACS Vantage, BD Biosciences) and seeded on the 96-well plates with serial dilution to obtain a single-cell-derived colony. Two weeks later, cells are collected to determine expression levels of MARCH5 protein using immunoblot.

### RNA interference

Human-specific siRNA for *MARCH5* (Supplementary Table 1) was custom-synthesized by Invitrogen. The siRNA targeting mouse *March5* was purchased from Thermo Scientific.

### Luciferase reporter assays

HEK293T cells were transfected with a mixture of luciferase reporter plasmid, renilla luciferase plasmid, an indicated variety expression plasmid or control (pcDNA) plasmid. Transfected cells were stimulated with PR8-GFP (1 multiplicity of infection), VSV-GFP (0.01 multiplicity of infection) viruses or poly(I:C) (1 μg μl^−1^). Luciferase activity was measured at 24–36 h after transfection using a luminometer (Promega) with a dual-luciferase reporter assay system according to the manufacturer's instructions (Promega). Data represent relative firefly luciferase activity, normalized to Renilla luciferase activity.

### Virus replication assay

To analyse viral replication, after cells were infected with VSV-GFP and PR8-GFP in serum-free media for 2 h, extracellular viruses were removed by replacing with complete media. The cultured media of virus-infected cells were collected and used for measurement of viral titre. Viral titre was determined in Vero cells by standard plaque assay. GFP-tagged VSV or H1N1 influenza virus (A/PR8/8/34) replication levels were measured with Fluorescence module of GloMax-Multi Microplate Multimode Reader (Promega). Fluorescence and phase-contrast images were acquired using a Nikon eclipse Ti with a 20 × 1.4 numerical aperture (NA) Plan-Apochromat objective. Images were captured with an iXonEM+897 Electron Multiplying charge-coupled device camera and analysed using the NIS elements Ar microscope imaging software.

### Type-I interferon and proinflammatory cytokine bioassay

To measure type-I interferon and proinflammatory cytokines, mouse IFN-β (PBL interferon source), mouse IL-6 (BD Biosciences), human IFN-β (PBL interferon source) and human IL-6 (BD Biosciences) ELISA kits were used. After infection of virus to cells, the levels of secreted cytokines were measured in supernatant of infected cell. For mice, plasma levels of type-I interferon and proinflammatory cytokines were analysed.

### *In vivo* and *in vitro* ubiquitination assay

Cells were treated with 10 μM of MG132 for 12 h before harvest. Whole cells were lysed with RIPA buffer (50 mM Tris-HCl (pH 7.4), 150 mM NaCl, 1% NP-40, 0.1% SDS, 0.1%s sodium deoxycholate, 5 mM EDTA and 5 mM EGTA) containing complete protease and phosphatase inhibitors. The same amount of protein lysates (700∼1,000 μg) was immunoprecipitated with anti-Flag or anti-MAVS antibody and further incubated with protein G agarose beads at 4 °C for 1 h 30 min. The immune complex was washed extensively four times with RIPA buffer and boiled for 5 min with 2 × sample buffer. Analysis of ubiquitylation was performed by immunoblotting using anti-Ub antibody. For *in vitro* ubiquitination assay, immunoprecipitated Myc-MARCH5^WT^ and Flag-MAVS^WT^ were prepared from lysates of HEK293T cells transfected with Myc-MARCH5^WT^ or Flag-MAVS^WT^, individually. Immunoprecipitates were incubated with 100 ng of E1 (Boston Biochem), 400 ng of E2 (UbcH5b, Boston Biochem) and 2 μg of ubiquitin (Boston Biochem) in reaction buffer (50 mM Tris (pH 7.4), 2 mM MgCl_2_, 4 mM ATP (Sigma), 1 mM dithiothreitol at 30 °C for 2 h, and the reaction was terminated by addition of 4 × sample buffer.

### Immunoblot analysis and immunoprecipitation assay

For immunoprecipitation assays, cells were lysed with RIPA buffer (50 mM Tris-HCl (pH 7.4), 150 mM NaCl, 1% NP-40, 0.1% SDS, 0.1% sodium deoxycholate, 5 mM EDTA, 5 mM EGTA) supplemented with protease and phosphatase inhibitors. Whole-cell lysates (500∼1,000 μg) were immunoprecipitated with 1 μg of indicated antibodies at 4 °C for 10–12 h with agitation. After further incubation with protein A-sepharose beads (GE Healthcare Bio-Science AB) for 1 h 30 min, the bead-bound immunoprecipitates were washed with RIPA buffer four times, and eluted with 2 × SDS sample buffer by boiling for 5 min. For immunoblotting analysis, cell lysates lysed by RIPA buffer were resolved using SDS–PAGE and transferred to the nitrocellulose membrane (Millipore). Immunoblots were visualized using the ECL system (Bio-Rad). The following antibodies were used in immunoblotting analysis: Anti-MARCH5 (1:1,000), anti-MAVS (1:1,000) and anti-IRF3 (1:1,000) were from Abcam. Anti-FLAG (M2; 1:2,000) and anti-Actin (1:2,000) were from Sigma. Antibodies for anti-HA (1:1,000), anti-Ub (1:1,000), anti-Tubulin (1:1,000), anti-GAPDH (1:1,000) and anti-c-Myc (1:1,000) were purchased from Santa Cruz Biotechnology. Anti-K48-Ub (1:1,000) and anti-K63-Ub (1:1,000) antibodies were from Millipore. Anti-phospho-IRF3 (Ser396; 1:1,000), anti-NF-κB p65 (1:1,000) and anti-phospho-NF-κB p65 (Ser536; 1:1,000) antibodies were purchased from Cell Signaling Technology.

### CHX chase assay

After transfection of plasmids, cells were treated with CHX (50 μg ml^−1^) for various times ranging from 0 to 24 h. Lysates were lysed and analysed by immunoblotting with anti-MAVS and GAPDH antibodies.

### Immunofluorescence

HEK293T cells seeded on coverslips in six-well plates were transfected with plasmids and incubated for 36 h. For visualization of mitochondria, cells were stained with 125 nM MitoTracker Red (Molecular Probes) for 30 min before harvest. Cells were fixed with 4% paraformaldehyde in PBS for 10 min at room temperature. Fixed cells were permeabilized with methanol for 20 min at −20 °C. For immunofluorescence staining, cells were blocked with 1% bovine serum albumin in PBS for 1 h at room temperature, followed by primary antibody incubation overnight at 4 °C. Cells were washed three times and incubated with fluorescence-conjugated secondary antibody for 1 h at room temperature. Fluorescence images were captured with an LSM 710 and analysed using the LSM 710 image software (Carl Zeiss) using 40 × 1.1 NA water immersion objective, controlled by the ZEN2011 programme (all from Carl Zeiss).

### Quantitative real-time PCR and RT–PCR

Total RNA was isolated from cultured cells and mouse tissues, using the RNeasy RNA extraction Mini Kit (Qiagen). One microgram of total RNA was used to synthesize cDNA with RT–PCR reagent Kit (Enzynomix). Quantitative PCR was performed with gene-specific primer sets (Supplementary Table 1) and Mygenie 96 thermal block (Bioneer) using the SYBR Green PCR Matster Mix. Real-time PCR was carried out using the CFX96 TouchTM Real-Time PCR Detection System (Bio-Rad). Data were normalized to the level of *GAPDH* expression in each individual sample.

### Statistical analysis

For each result, error bars represent the mean±s.e.m. from at least three independent experiments. Statistical significance was performed with two-sided unpaired Student's *t*-test. *P* values are indicated in the legends.

### Ethics statement

The research protocols for the use of mice in this study were conducted following approval from the Institutional Animal Care and Use Committee of Bioleaders Corporation (Reference number BLS-ABSL-14-012). Animal experiments were conducted in biosafety level BSL-2 and BSL-3^+^ laboratory facilities.

## Additional information

**How to cite this article:** Yoo, Y.-S. *et al*. The mitochondrial ubiquitin ligase MARCH5 resolves MAVS aggregates during antiviral signalling. *Nat. Commun.* 6:7910 doi: 10.1038/ncomms8910 (2015).

## Supplementary Material

Supplementary Information Supplementary Figures 1-8 and Supplementary Table 1
