# Supplementary material for: The mitochondrial ubiquitin ligase MARCH5 resolves MAVS aggregates during antiviral signalling
Source: Nat Commun. 2015 Aug 6;6:7910. doi: 10.1038/ncomms8910 (PMC4918326; doi:10.1038/ncomms8910)
Supplement: Supplementary Information — Supplementary Figures 1-8 and Supplementary Table 1 [file ncomms8910-s1.pdf]

Supplementary Figure 1

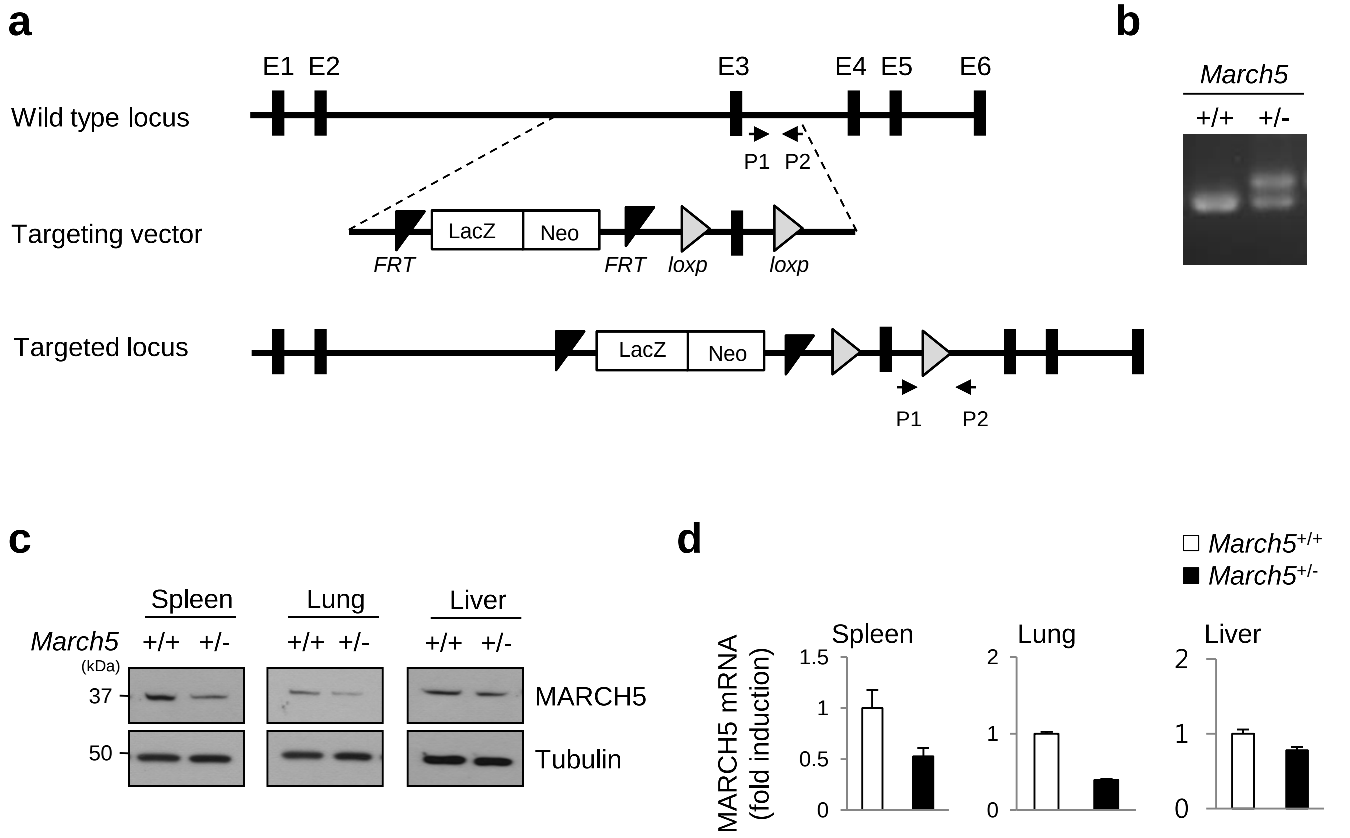

Supplementary Figure 1. Generation of *March5*$^{+/-}$ mice.

(a) A schematic drawing of the *March*5 locus and its targeting vector. C57BL/6 *March5*^{tm1a} mice harboring *Lox*P sites flanking *March5* exon 3 were generated at the European Mouse Mutant Archive (EMMA) and placed in the same genetic background. **(b)** PCR genotyping of *March5*$^{+/+}$ and *March5*$^{+/-}$ mice littermates using P1 and P2 primers. **(c)** Immunoblot analysis of MARCH5 protein expression levels in the spleen, lung, and liver of *March5*$^{+/+}$ and *March5*$^{+/-}$ mice. **(d)** Quantitative PCR analysis of *March5* mRNA expression in spleen, lung and liver from *March5*$^{+/+}$ and *March5*$^{+/-}$ mice. Error bars, mean ± SEM (n=3). All data are representative of at least three independent experiments.

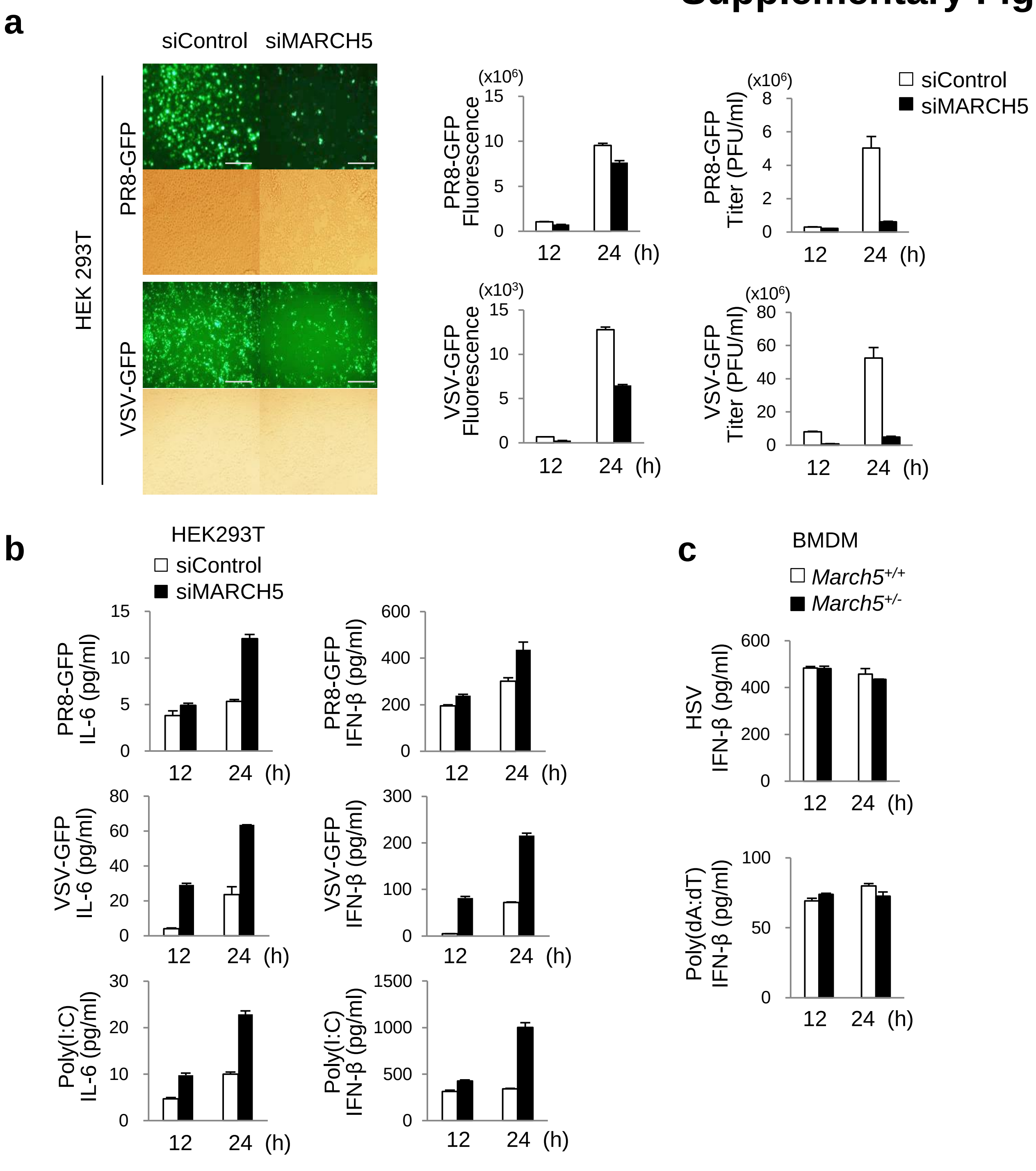

Supplementary Figure 2. MARCH5 depleted cells induced type-I interferon and proinflammatory cytokines by RNA viruses.

(a) Fluorescence microscopy analysis (left) and viral titer (right) by fluorescence analysis or plaque-assays after infection for 24 hours with VSV-GFP or PR8-GFP in MARCH5 siRNA expressing HEK293T cells. Error bars, mean ± SEM (n=3). Scale bars, 100 μm. **(b)** Bioassay of IL-6 or IFN-β (ELISA) in supernatants of MARCH5 siRNA expressing HEK293T cells. Cells were transfected with indicated siRNA, followed by infection of virus (PR8-GFP or VSV-GFP) or transfection with poly(I:C) for 24 hours. Error bars, mean ± SEM (n=3). **(c)** Bioassay of IFN-β (ELISA) in supernatants of *MARCH5*$^{+/+}$ and *MARCH5*$^{+/-}$ derived BMDM cells infected with HSV or transfected with poly(dA:dT) for indicated times. Error bars, mean ± SEM (n=3). All data are representative of at least three independent experiments.

Supplementary Figure 3

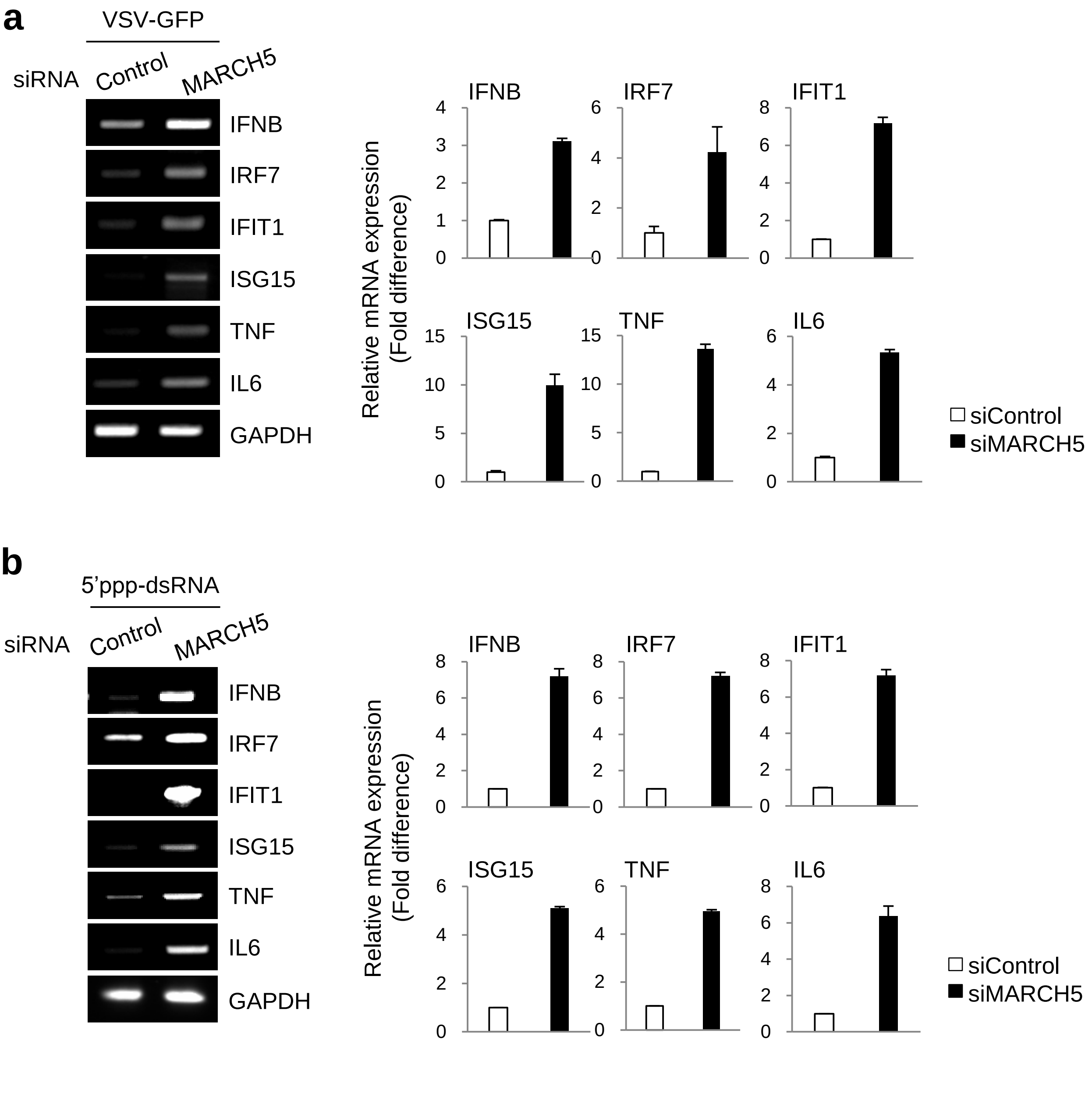

Supplementary Figure 3. Expression of IFN response genes are elevated in MARCH5-knockdown cells.

(a,b) Messenger RNA levels of IFNB, IRF7, IRIT1, ISG15, TNF, IL6, GAPDH in control or MARCH5 siRNA expressing HEK293T cells by RT-PCR analysis. Graphs represent fold changes of mRNA levels of indicated genes value were relative to control siRNA expressing cells, and those were normalized by GAPDH. Data represent the average of three independent experiments. Control or *MARCH5* siRNA expressing cells were infected with VSV-GFP (a) or transfected with 5'ppp-dsRNA (b) for 24 hours, followed by RT-PCR. Error bars, mean ± SEM (n=3). All data are representative of at least three independent experiments.

Supplementary Figure 4

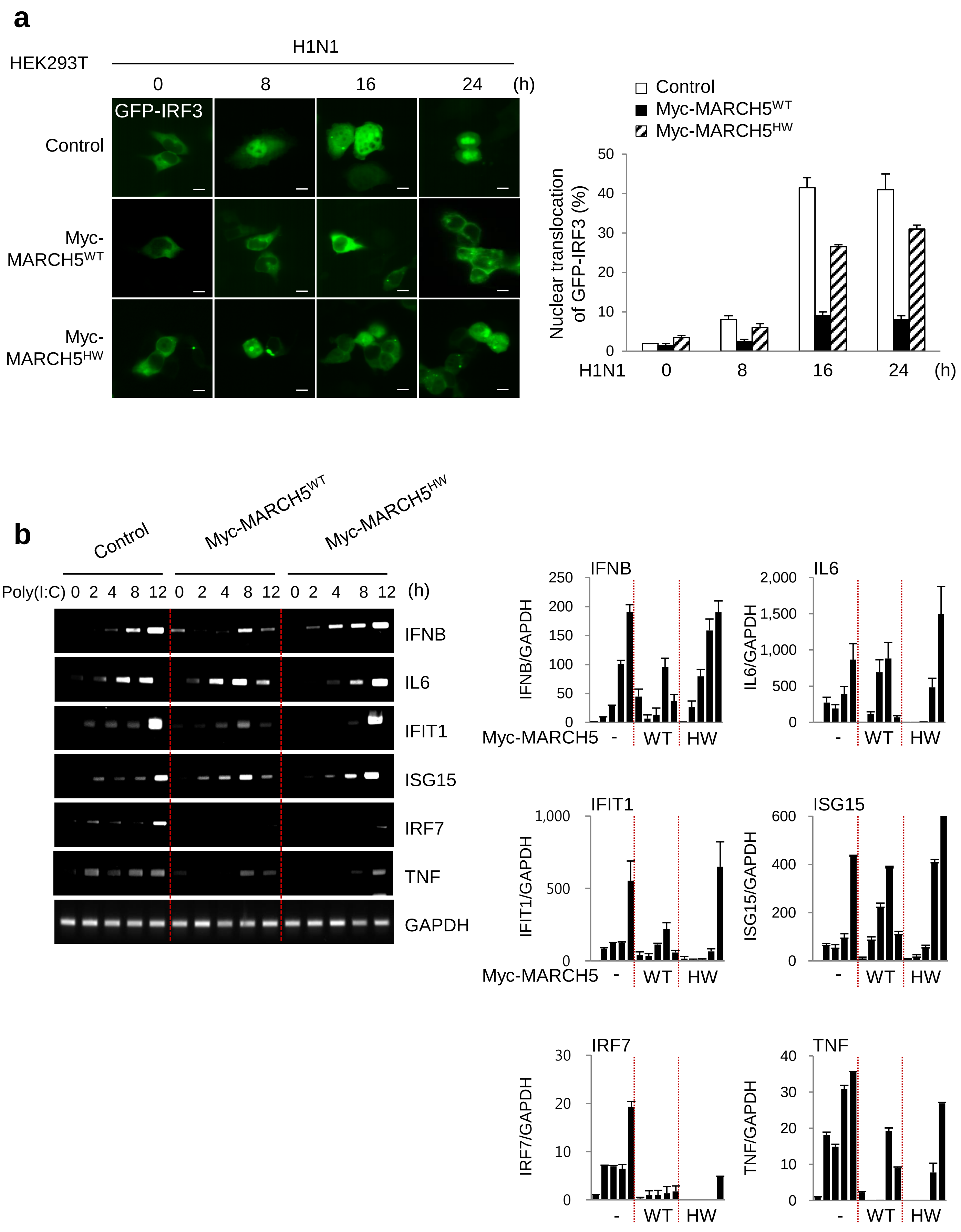

Supplementary Figure 4

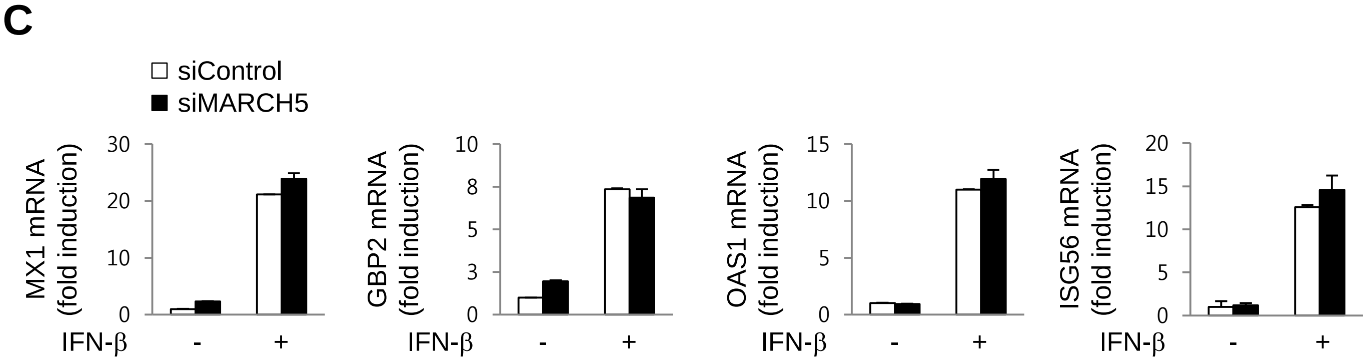

Supplementary Figure 4. MARCH5 negatively regulates type-I interferon signaling in an E3 ligase activity-dependent manner.

(a) Confocal microscopy analysis of GFP tagged IRF3 localization in HEK293T cells. Cells were co-expressed with Myc-MARCH5 (WT or H43W) and GFP-IRF3 for 24 hours, followed by infection with H1N1 virus for indicated times. Images of representative fields were captured by confocal laser microscopy. Scale bars represent 10 μm (upper). Graph represents quantification of GFP-IRF3 nuclear translocation. Population represents the percentage of cell population with nuclear translocation of GFP-IRF3. At least 100 cells in several fields were counted in three independent experiments. Error bars, mean ± SEM (n=3). **(b)** Messenger RNA expression levels of *IFNB*, *IL6*, *IFIT1*, *ISG15*, *IRF7, TNF* by RT-PCR analysis in Myc-MARCH5 WT or H43W overexpressing HEK293T cells with stimulation by poly(I:C) for indicated times. Graphs represent quantification of mRNA levels of indicated genes. Fold changes of mRNA of indicated genes value were relative to control cells, and those were normalized by GAPDH. Data represent the average of three independent experiments. Error bars, mean ± SEM (n=3). **(c)** mRNA expression levels of MX1, GBP2, OAS1, ISG56 in control or MARCH5 siRNA expressing Raw 264.7 cells by quantitative PCR analysis. Control or MARCH5 siRNA expressing cells were treated with IFN-β as indicated for 12 hours. Error bars, mean ± SEM (n=3). All data are representative of at least three independent experiments.

Supplementary Figure 5

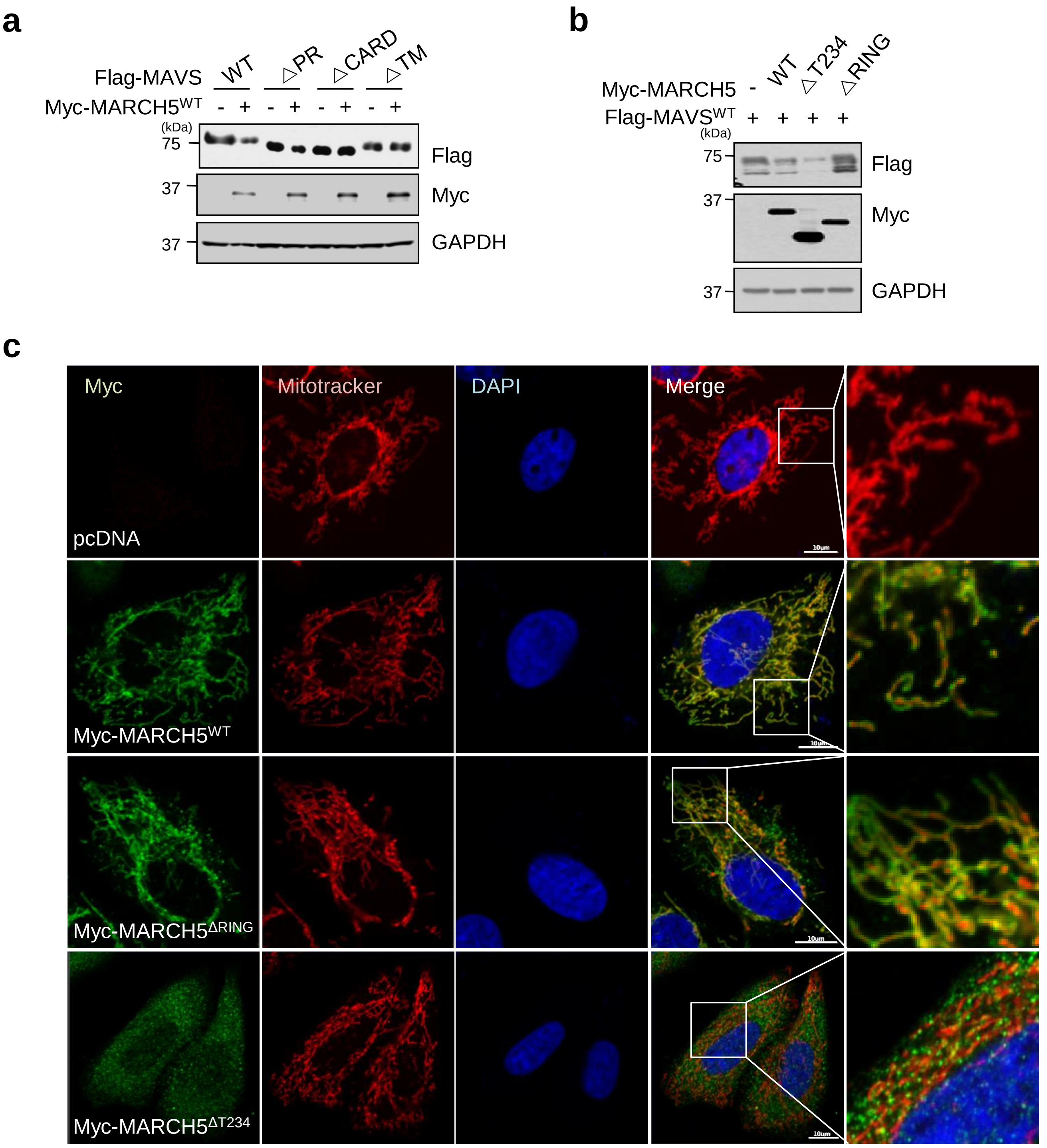

Supplementary Figure 5. RING of MARCH5 and CARD/TM of MAVS are important for their association.

(a) Immunoblot analysis of Flag-MAVS WT or truncated mutants expression levels in HEK293T cells transfected with or without Myc-MARCH5^{WT}. See full blots in supplementary Fig.8. **(b)** Immunoblotting of Flag-MAVS expression levels in HEK293T cells transfected with Myc-MARCH5^{WT} or truncated mutants. **(c)** Confocal immunofluorescence microscopy of Myc-MARCH5^{WT}, Myc-MARCH5$^{\triangle RING}$ or Myc-MARCH5$^{\triangle T234}$ in HeLa cells. All data are representative of at least three independent experiments.

Supplementary Figure 6

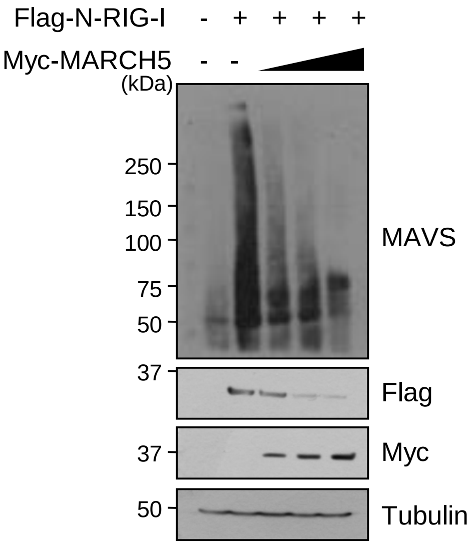

Supplementary Figure 6. The reduction of MAVS aggregates by MARCH5 overexpression.

Immunoblot analysis of oligomeric MAVS in HEK293T cells. Cells were transfected with Flag-N-RIG-I with Myc-MARCH5^{WT} dose dependent manner. Lysates were mixed with or without 6X native sample buffer, followed by SDS-PAGE. All data are representative of at least three independent experiments. See full blots in supplementary Fig.8.

Supplementary Figure 7

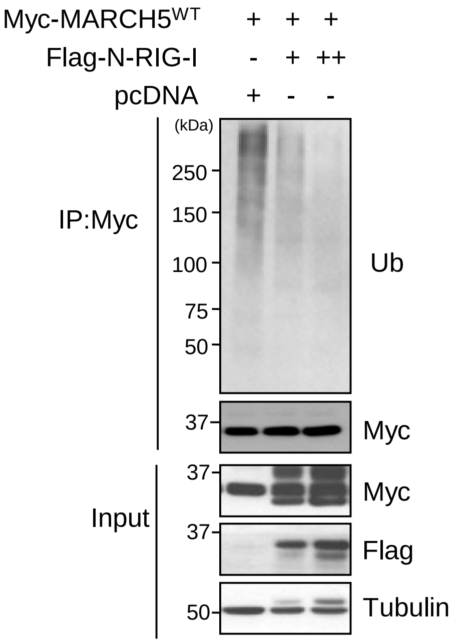

Supplementary Figure 7. Autoubiquitination of the MARCH5 proteins.

Immunoblot analysis of MARCH5 autoubiquitination in HEK293T cells. Cells were transfected with Myc-MARCH5^{WT} with Flag-N-RIG-I dose dependent manner. Myc-tagged MARCH5 proteins were immunoprecipitated with anti-Myc antibodies. Immunoblot of autoubiquitination assay for Myc-tagged MARCH5. All data are representative of at least three independent experiments. All data are representative of at least three independent experiments**.** See full blots in supplementary Fig.8.

Supplementary Figure 8

Figure 4-c

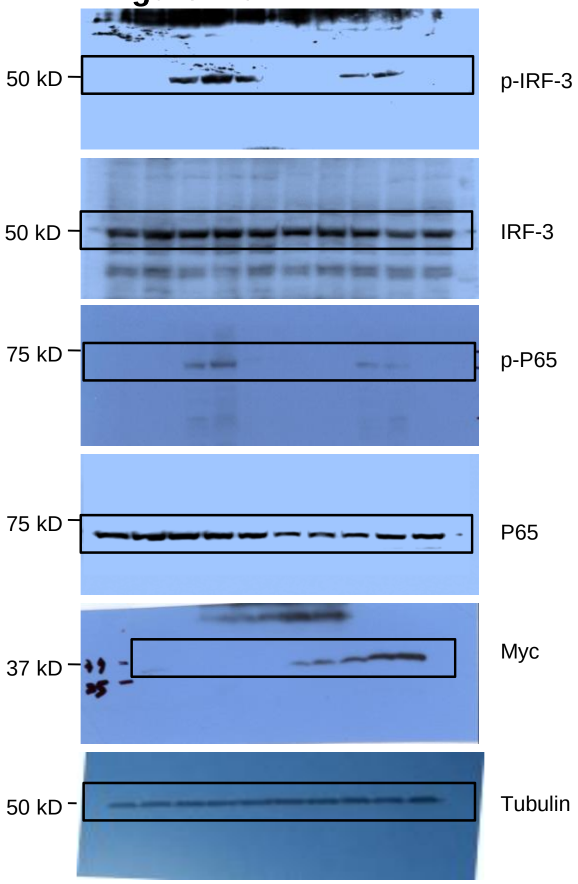

Figure 5-a

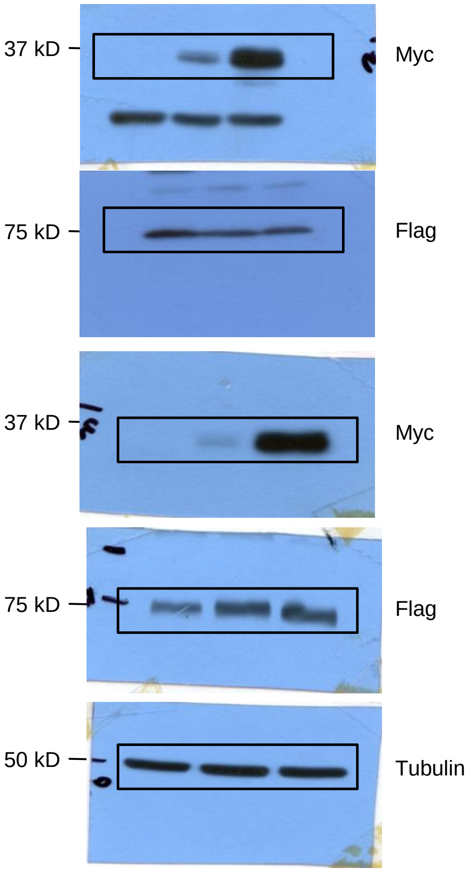

Figure 5-c

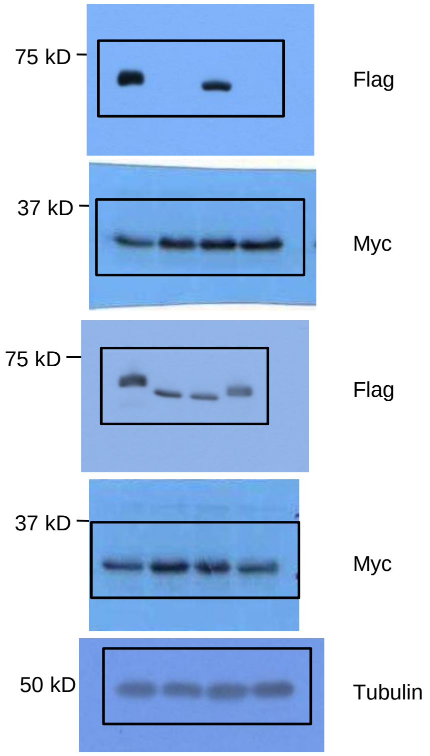

Figure 5-d

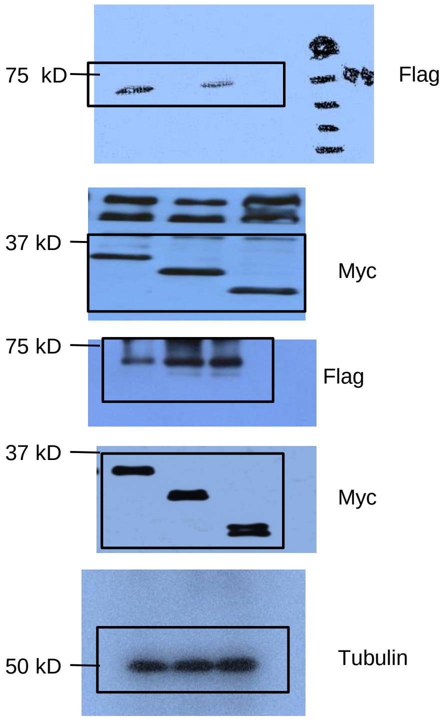

Figure 5e

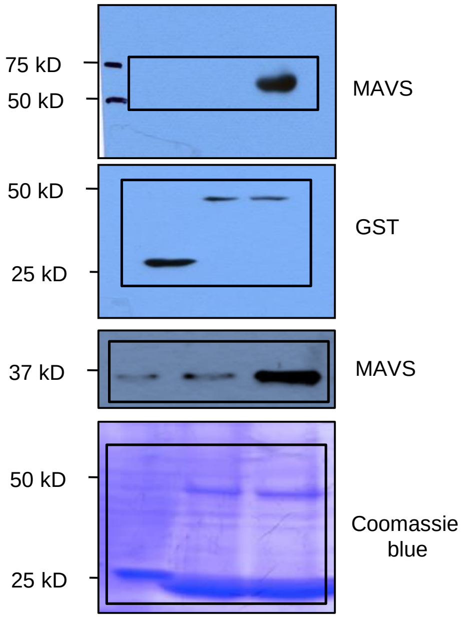

Supplementary Figure 8

Figure 5-f

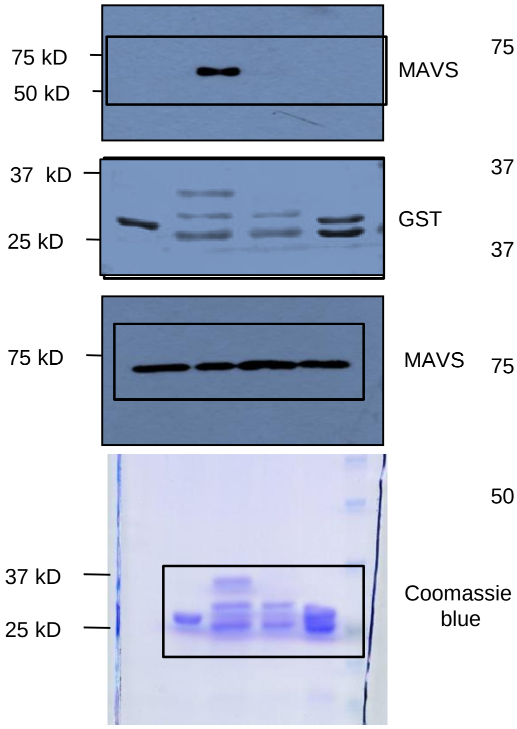

Figure 6-a

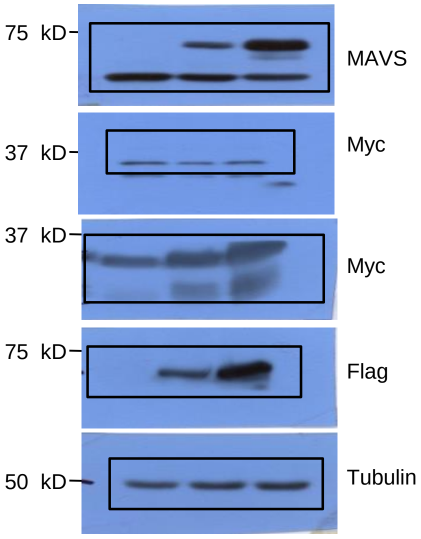

Figure 6-b

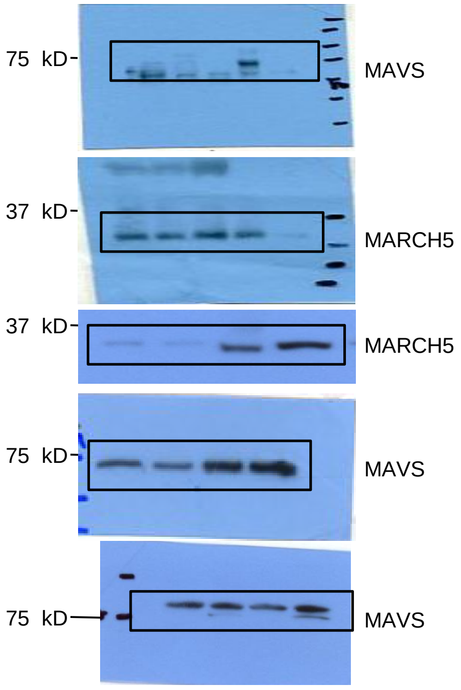

Figure 6-c

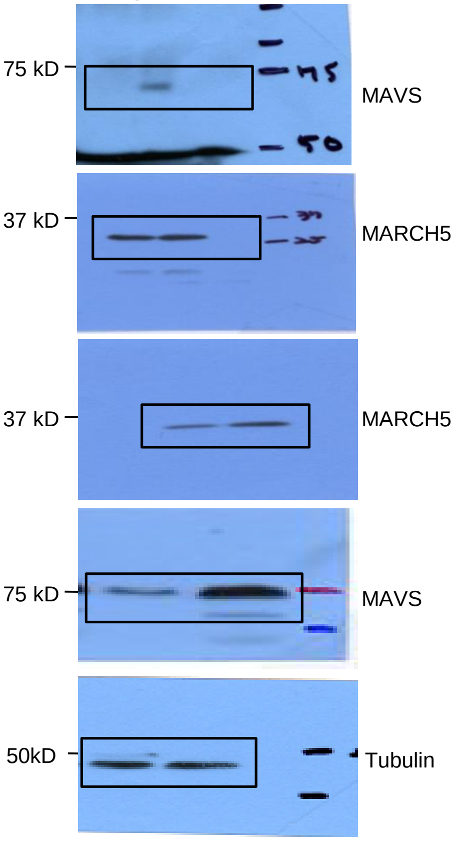

Figure 6-d

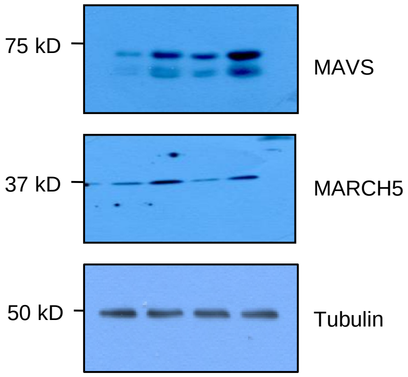

Figure 6-e

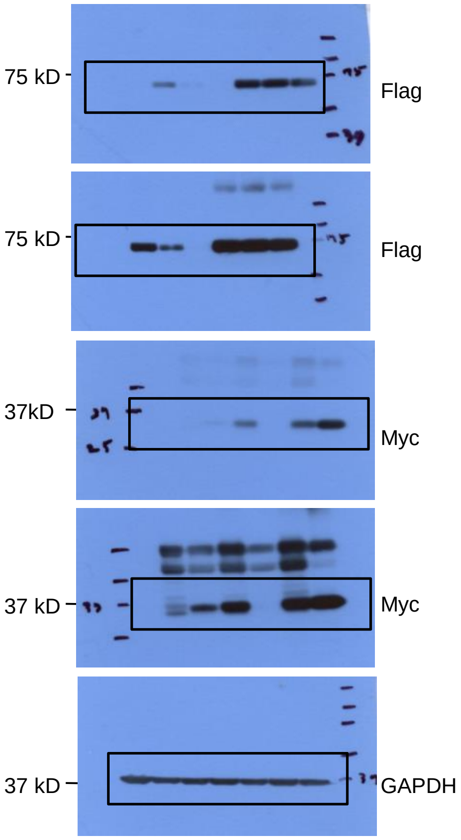

Supplementary Figure 8

Figure 6-f

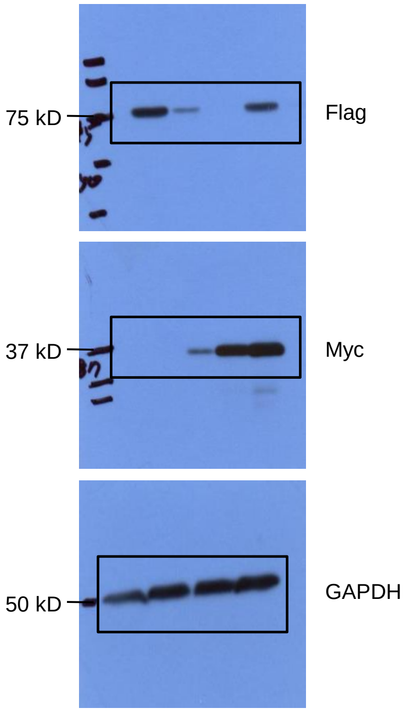

Figure 6-g,h

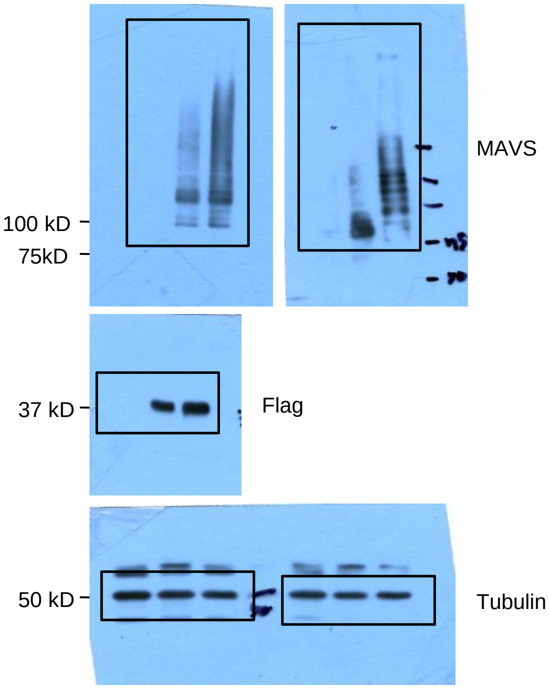

Figure 6-i

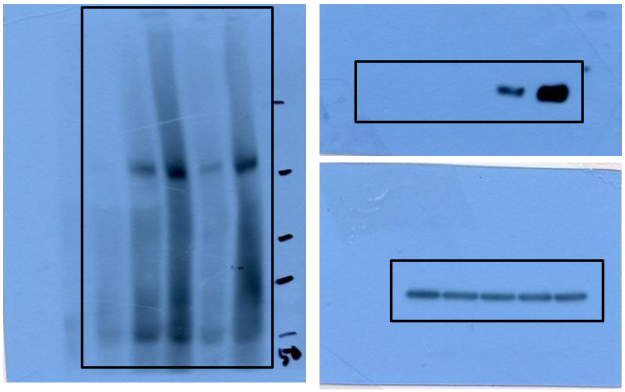

Figure 6-j

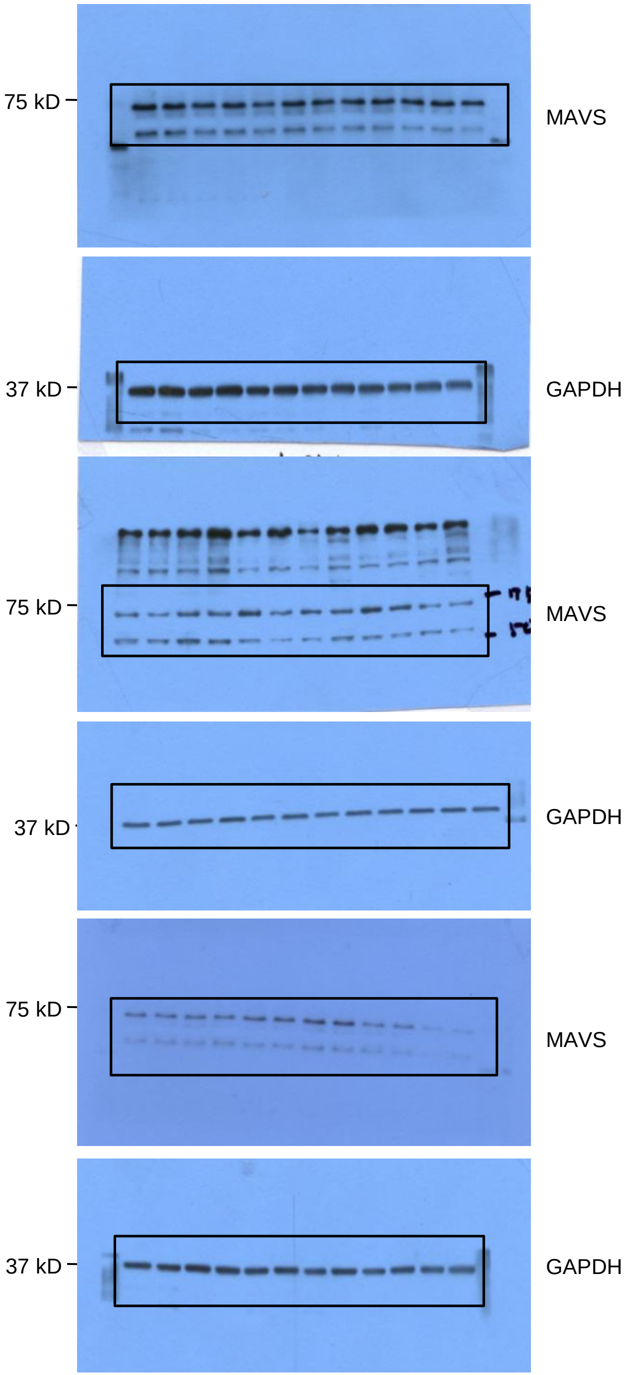

Supplementary Figure 8

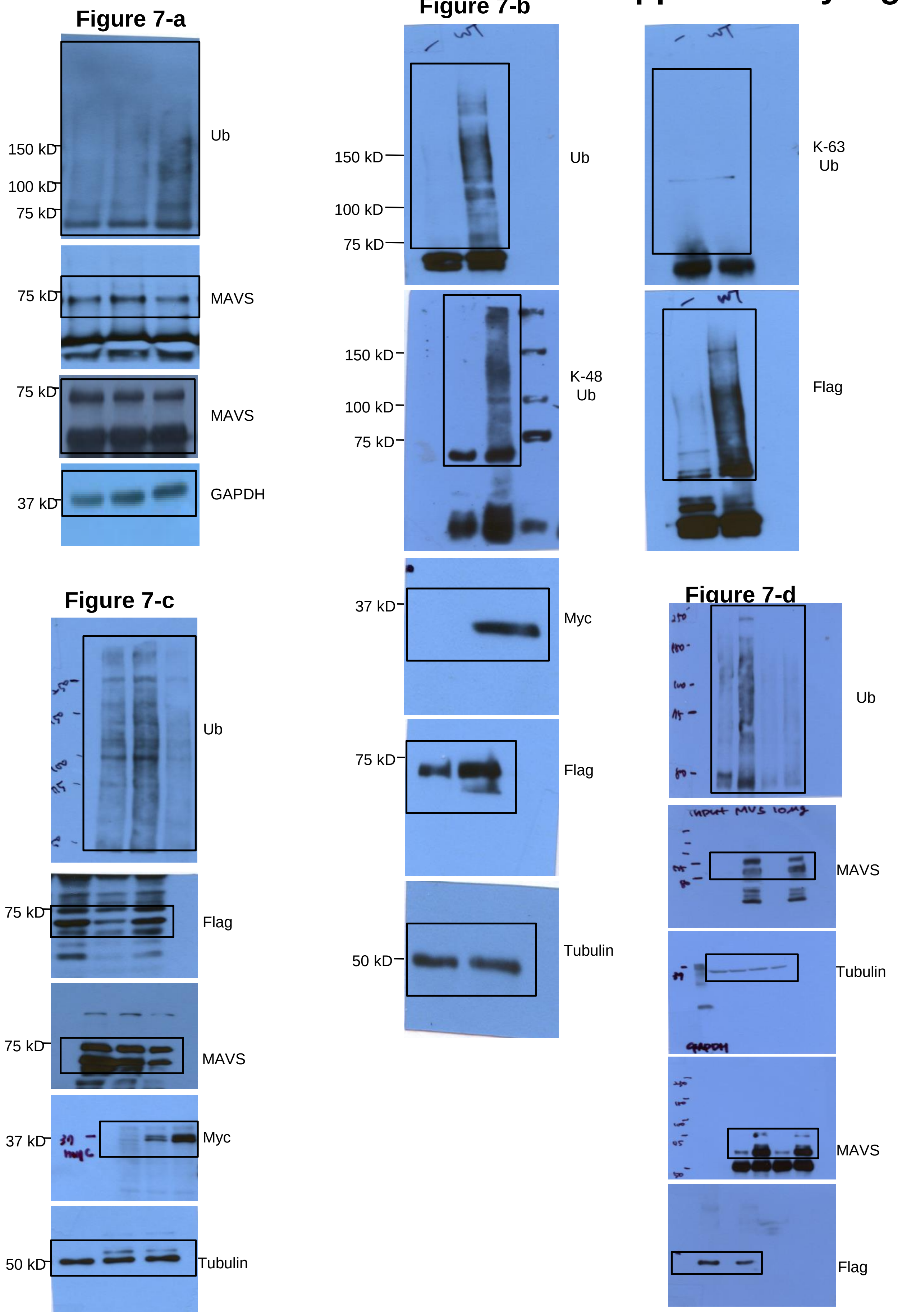

Supplementary Figure 8

Figure 7-e

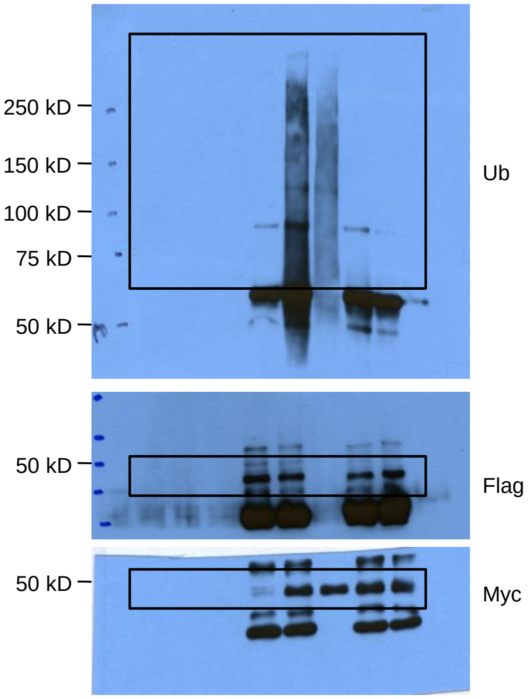

Figure 8-a

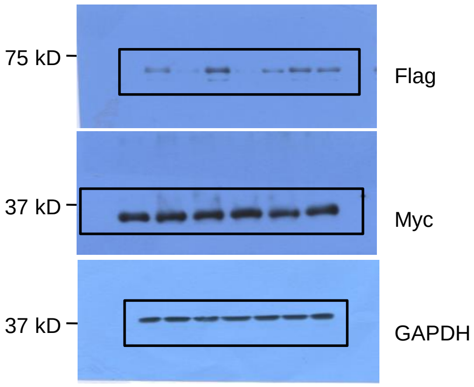

Figure 8-b

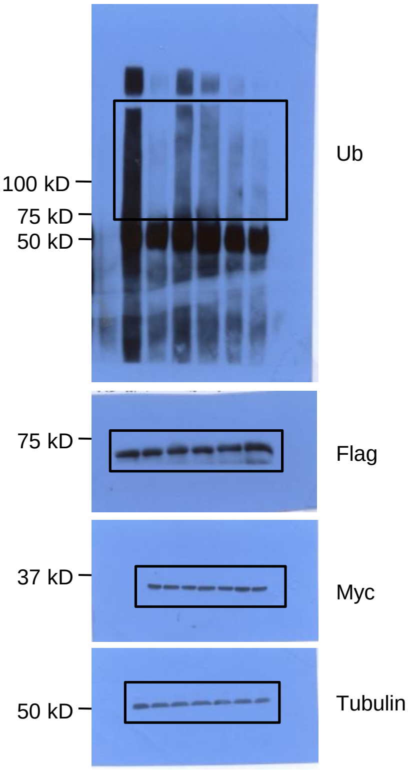

Supplementary Figure 8

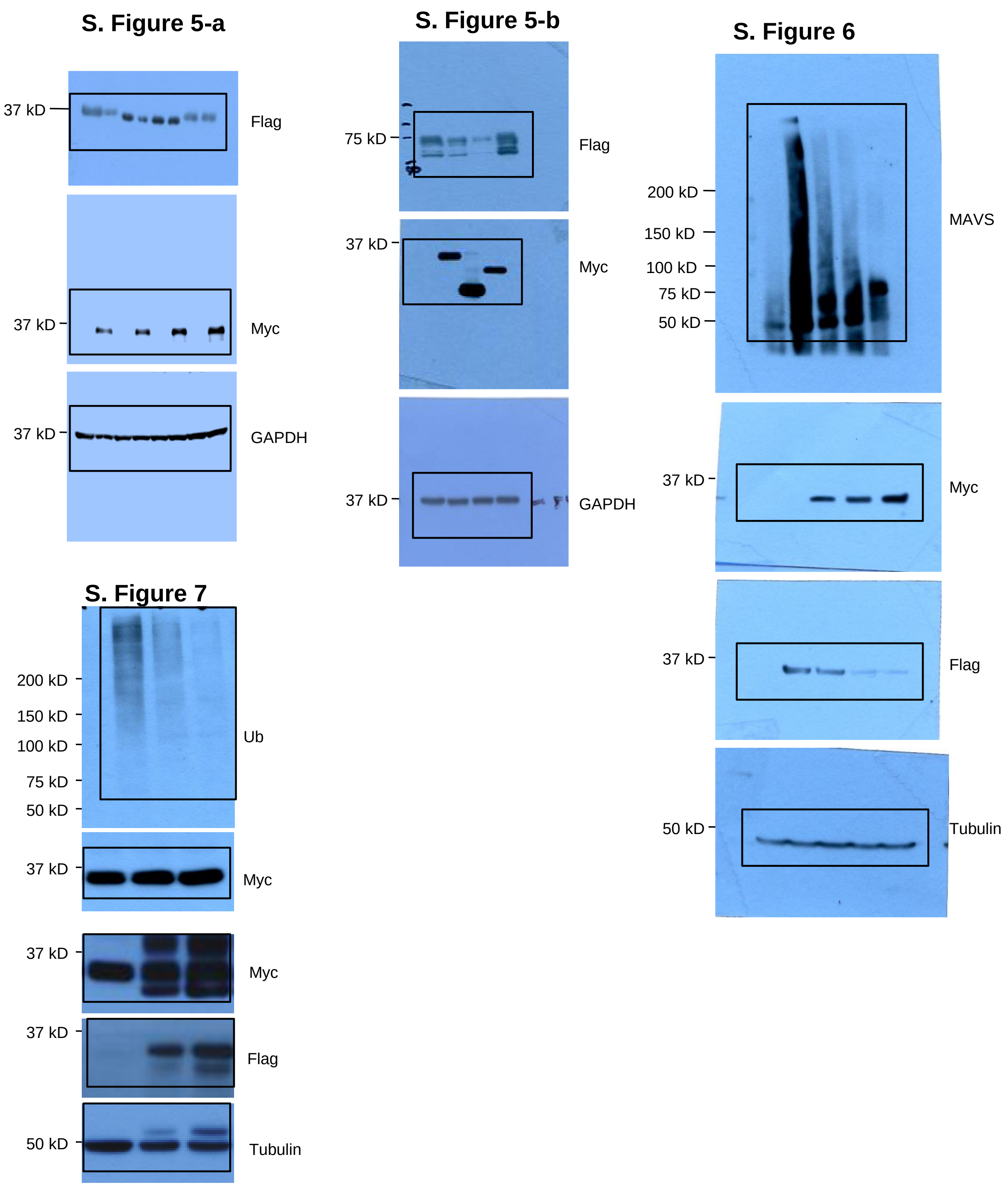

Supplementary Figure 8. Full blots for immunoblotting and GST-pull down assay shown in figures and supplementary figures.

RT QPCR primer (Mouse)	Forward primer	Reverse Primer
Ifnb1	TCCAAGAAAGGACGAACATTCG	TGCGGACATCTCCCACGTCAA
Ifit1	AGAGAACAGCTACCACCTTT	TGGACCTGCTCTGAGATTCT
Gbp2b	AAAAACTTCGGGGACAGCTT	CTGAGTCACCTCATAAGCCAAA
Oas1b	GAGGCGGTTGGCTGAAGAGG	GAGGAAGGCTGGCTGTGATTGG
Mx1	ACAAGCACAGGAAACCGTATCAG	AGGCAGTTTGGACCATCTTAGTG
Eif2ak2	GCCAGATGCACGGAGTAGCC	GAAAACTTGGCCAAATCCACC
Isg15	CAATGGCCTGGGACCTAAA	CTTCTTCAGTTCTGACACCGTCAT
Il6	TCCATCCAGTTGCCTTCTTGG	CCACGATTTCCCAGAGAACATG
RT QPCR primer (Human)	Forward primer	Reverse Primer
IFNB	ATGAAGATCTCTGCAGCTGCC	TAGGCAAAGCAGCAGGGAGTG
IRF3	AGAGGCTCGTGATGGTCAAG	AGGTCCACAGTATTCTCCAGG
IFIT1	CACAGTGATGCTAGTGGTAC	AGGTTGTGTATTCCCACACTGTA
ISG15	GCGAGATCACCCAGAAGATT	GCCCTTGTTATTCCTCACCA
TNF	ATGTGCTCCTCACCCACACC	CCCTTCTCCAGCTGGAAGAC
IRF7	CCGCCCAAACATGCAAGATGGC	CCGCCCAAACCAGGTGTCACG
IL6	CTACATTGCCGAAGAGCCCTC	CCACACAGACAGCCACTCACC

hMARCH5 siRNA	GGGUGGAAUUGCGUUUGUUTT

Supplementary Table 1. QPCR primer sequences and hMARCH5 siRNA sequences
